# The original and two new derivative versions of the COMPERA 2.0 risk assessment model: useful tools for guiding balloon pulmonary angioplasty

**DOI:** 10.1186/s12931-022-02232-1

**Published:** 2022-11-15

**Authors:** Yi Zhang, Xin Li, Qi Jin, Qin Luo, Qing Zhao, Tao Yang, Qixian Zeng, Lu Yan, Anqi Duan, Zhihua Huang, Meixi Hu, Changming Xiong, Zhihui Zhao, Zhihong Liu

**Affiliations:** 1 Center for Pulmonary Vascular Diseases, Fuwai Hospital, National Center for Cardiovascular Diseases, Chinese Academy of Medical Sciences and Peking Union Medical College, No.167 Beilishi Rd, Xicheng District, Beijing, 100037 China; 2 Department of Cardiology, Shanghai Institute of Cardiovascular Diseases, Zhongshan Hospital, Fudan University, Shanghai, China

**Keywords:** Chronic thromboembolic pulmonary hypertension, Balloon pulmonary angioplasty, Risk stratification

## Abstract

**Background:**

The COMPERA 2.0 4-stratum (4-S) risk score has been demonstrated superior over the 3-stratum (3-S) one in patients with pulmonary arterial hypertension and medically managed patients with chronic thromboembolic pulmonary hypertension (CTEPH). We aimed to determine the prognostic value of the original 4-S and 3-S COMPERA 2.0 risk score and two new derivative versions in CTEPH patients who underwent balloon pulmonary angioplasty (BPA).

**Methods:**

We retrospectively enrolled 175 BPA-treated patients with CTEPH. We assessed the risk stratification before and after each BPA session of CTEPH patients by the original 4-S and 3-S COMPERA 2.0 risk score (by rounding decimal to the nearest integer) and two new proposed derivative versions: the modified version (by rounding decimal to the next integer) and a hybrid version that fuses the original and modified versions. The primary endpoint was clinical worsening events. The secondary outcomes were achieving low-risk profile and mean pulmonary arterial pressure (mPAP) < 30 mmHg at follow-up. We used the Kaplan–Meier curve analysis to assess the survival differences between stratified patients. The comparative model’s performance was evaluated in terms of discrimination by Harrell’s C-index.

**Results:**

All versions of COMPERA 2.0 4-S model outperformed the 3-S one in discriminating the differences in echocardiographic and hemodynamic parameters and clinical worsening-free survival rates. The original and hybrid 4-S model could independently predict the primary and secondary endpoints, and the hybrid version seemed to perform better. The first BPA session could significantly improve risk profiles, and these changes were associated with the likelihood of experiencing clinical worsening events, achieving a low-risk profile and mPAP < 30 mmHg at follow-up. The number of BPA sessions required to achieve low risk/mPAP < 30 mmHg increased as the baseline risk score escalated.

**Conclusions:**

The COMPERA 2.0 4-S model outperformed the 3-S one in BPA-treated patients with CTEPH. The 4-S model, especially its hybrid version, could be used to predict clinical outcome before the initiation of BPA and monitor treatment response.

**Supplementary Information:**

The online version contains supplementary material available at 10.1186/s12931-022-02232-1.

## Introduction

Risk stratification is integrated throughout the management of pulmonary hypertension. The 2015 European guidelines, together with other risk assessment tools such as the Swedish Pulmonary Arterial Hypertension Registry (SPAHR), classified patients as low-, intermediate- and high risk, with estimated 1-year mortality rates of < 5%, 5–10% and > 10%, respectively [1–5]. Previous studies showed that approximately 60–70% patients with pulmonary arterial hypertension had intermediate risk profile both at baseline and follow-up [2, 4], and a more granular risk prediction model that could better reflect patients’ baseline characteristics and response to treatment may be more beneficial to this group. In an exploratory analysis of SPAHR, Kylhammar et al. further divided the patients in the intermediate risk group into a low-intermediate (risk score 1.5–1.99) and a high-intermediate (risk score 2.0–2.4) risk group. They showed that patients at intermediate-low risk had better survival rates than those at intermediate-high risk leading to a better characterization of the intermediate risk group [6]. Immediately after, Hoeper et al. developed COMPERA 2.0 risk score, by using 3 noninvasive variables, namely World Health Organization functional class (WHO-FC), 6 min walk distance (6MWD), and N-terminal pro-brain natriuretic peptide (NT-proBNP)/brain natriuretic peptide (BNP) [7]. The authors reported that the 4-stratum (4-S) model outperformed the 3-stratum (3-S) model in predicting prognosis and reflecting treatment response, which was further externally validated by Boucly et al. [8].

Recently, Stubbs et al. [9] validated the 3-S and 4-S COMPERA 2.0 risk stratification in medically managed patients with CTEPH. Unfortunately, its prognostic value in patients with CTEPH who underwent balloon pulmonary angioplasty (BPA) remains unclear. We aimed to determine the prognostic value of the original 3-S and 4-S COMPERA 2.0 risk score and two new derivative versions in CTEPH patients who underwent BPA.

We hypothesized that the original COMPERA 3-S and 4-S prediction models could predict clinical outcome of BPA-treated patients with CTEPH and that the new derivative corresponding versions could further improve the discriminatory power.

## Materials and methods

### Study design and participants

This was a retrospective cohort study, conducted at Fuwai Hospital, Chinese Academy of Medical Sciences (Beijing, China). The study protocol was approved by the Ethics Committee of Fuwai Hospital. Written informed consent was obtained from each patient. We reviewed medical charts of all BPA-treated patients with CTEPH from May 2018 to October 2021. The diagnosis of CTEPH was based on current European guidelines [1]. Eligibility for BPA was jointly assessed by an expert CTEPH team, including pulmonary endarterectomy surgeons, pulmonary hypertension radiologists and pulmonary hypertension specialists. The following patients were excluded from the current study: (1) Missing any variables of interest (i.e., WHO-FC, 6MWD and NT-proBNP); (2) BPA procedure-related in-hospital death (3) Missing follow up data; and (4) Undergoing pulmonary endarterectomy at follow-up. Data collection was performed through electronic chart review.

### Risk stratification

According to the previously described cutoff points for WHO, 6MWD and NT-proBNP (Additional file 3: Tables S1 and S2) [7, 8], we assigned 1 to 4 points to each variable. The risk score for each patient was calculated as follows: sum of all points/number of variables. Then, based on the methods of dealing with decimal, we used 3 versions of COMPERA 2.0 risk score to classify patients, namely the original version, modified version, and hybrid version. The original 3-S and 4-S COMPERA 2.0 risk score rounds decimal to the nearest integer; the modified version rounds decimal to the next integer; the hybrid version fuses the original and modified versions (risk score < 2 in 3-S and < 3 in 4-S, rounds decimal to the nearest integer; risk score ≥ 2 in 3-S and ≥ 3 in 4-S, rounds decimal to the next integer)(Table 1).

**Table 1:** Values of 3-S and 4-S COMPERA 2.0 risk score versions

3-S versions	Low risk	Intermediate risk	High risk
Original 3-S version	1.0–1.4	1.5–2.4	2.5–3.0
Modified 3-S version	1.0	1.1–2.0	2.1–3.0
Hybrid 3-S version	1.0–1.4	1.5–2.0	2.1–3.0

4-S versions	Low risk	InterM-low risk	InterM-high risk	High risk
Original 4-S version	1.0–1.4	1.5–2.4	2.5–3.4	3.5–4.0
Modified 4-S version	1.0	1.1–2.0	2.1–3.0	3.1–4.0
Hybrid 4-S version	1.0–1.4	1.5–2.4	2.5–3.0	3.1–4.0

3-S, 3-Stratum; 4-S, 4-Stratum

Risk stratification was performed within 7 days prior to and after each BPA session. Therefore, risk stratification estimation accompanies the BPA procedure. In our center, patients usually return for the subsequent BPA assessment approximately every 3 months. The median time interval between two BPA sessions was 3 (1.25, 5) months.

### Balloon pulmonary angioplasty

Balloon pulmonary angioplasty was performed as we previously described and the detailed procedure was provided in the Additional file 1 [10]. Briefly, right heart catheterization was firstly performed to measure baseline hemodynamics before each BPA session. Then, pulmonary angiography was performed to acquire an overall view of the filling defect. Based on selective pulmonary angiography, a 0.014-inch guidewire was passed across the selected lesion. Subsequently, an appropriately sized balloon was carefully inflated with attention to vessel size and balloon behavior. Hemodynamics was measured again when each BPA session was completed.

### Clinical outcome

After discharge, patients were regularly followed up by telephone call and/or outpatient/inpatient examination. The primary outcome was clinical worsening, which was defined as all-cause death, unplanned PH-related hospitalization, unsatisfactory long-term clinical response (NT-proBNP increased by 100% or to ≥ 1800 ng/L from baseline to follow-up) [11, 12], and appearance or worsening of signs/symptoms of right heart failure. Time to clinical worsening was defined as the time from the date of the first BPA session to the first occurrence of clinical worsening events or the end of follow-up (2021-12-25).

There were two secondary outcomes. The first was achieving the low-risk criteria of the original/hybrid COMPERA 2.0 risk score after the first BPA session or during subsequent BPA sessions. The other secondary outcome was positive hemodynamic response during subsequent BPA sessions, which was defined as mean pulmonary arterial pressure (mPAP) < 30 mmHg (alternative threshold used in many centers) [13].

### Statistical analysis

Continuous variables are expressed as mean ± standard deviation or median (interquartile range). Categorical variables are given as counts (percentages). Comparison among groups was performed using one-way ANOVA, Kruskal–Wallis tests, or the *Chi*-square test, as appropriate. Spearman correlation coefficient was used to assess the correlation between the grade of COMPERA 2.0 risk score and other variables. Kaplan–Meier curve analysis with log rank test was used to evaluate the survival differences between stratified patients. The associations between COMPERA 2.0 risk score and clinical worsening were determined by using Cox regression model. Variables with *P* < 0.05 in univariable analysis or of clinical significance were selected for multivariable Cox regression (enter method). We used Harrell’s C-index to assess the discriminatory power among the different versions of the COMPERA 2.0 risk stratification. The associations between COMPERA 2.0 risk score and achieving low risk/positive hemodynamic response at follow-up were determined by using logistic regression model. Variables with *P* < 0.05 in univariable analysis or of clinical significance were selected for multivariable logistic regression (enter method). Also, the event-per-variable ratio in each multivariable regression was assured ≥ 5:1. A two-sided *P* value of < 0.05 indicated statistical significance. Data were analyzed using SPSS 26.0 (IBM SPSS Corp.; Armonk, NY, USA) and Prism GraphPad 9 (GraphPad Software, LA Jolla, CA, USA).

## Results

### Patient enrollment

One hundred and eighty-four patients with CTEPH underwent BPA from May 2018 to October 2021. Among these patients, 9 patients were excluded: missing 6MWD (n = 3), lost to follow-up (n = 3), undergoing pulmonary endarterectomy at follow-up (n = 2), in-hospital death due to reperfusion pulmonary edema during the first BPA session (n = 1). Ultimately, 175 patients (92 females, mean age 60.1 ± 10.8 years) were included in the current study. The indications for performing BPA were: refused pulmonary endarterectomy (n = 8); persistent pulmonary hypertension post pulmonary endarterectomy (n = 2); high risk–benefit ratio (n = 12); and inaccessible distal lesion (n = 153). During the study period, a total of 504 BPA sessions performed [median (interquartile range): 3 (2, 4)/per person], with 3792 pulmonary vessels dilated [median (interquartile range): 20 (10, 30)/per person]. Among included 175 patients, 125 patients returned for the assessment of further BPA sessions and underwent reevaluation right heart catheterization. The median time interval from the first BPA session to the latest one was 9 (3, 18) months.

### Risk stratification at baseline and clinical worsening

**Fig. 1:**
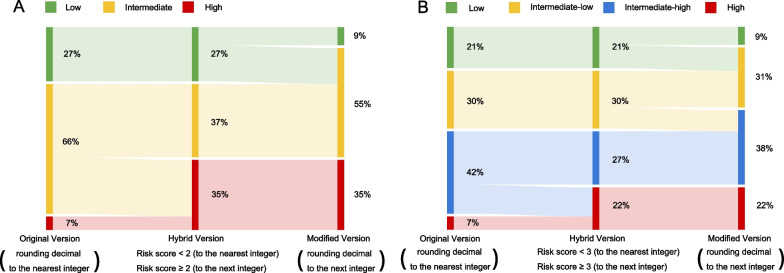
Sankey diagrams showing changes in risk profiles when the original COMPERA 2.0 risk score was converted to its derivative versions. Each panel shows the flow of patients between risk strata (nodes) from the original version to its derivative versions. The width of each band is weighted to the proportion of patients who had a given trajectory. **A** The 3-stratum model. **B** The 4-stratum model

The baseline characteristics of all included patients, stratified by risk stratum, were presented in Table 2 and Additional file 3: Tables S3, S4. The proportion of low, intermediate-low, intermediate-high, and high risk, in the original 4-S COMPERA 2.0 risk score, were 21%, 30%, 42% and 7%, respectively (Table 2 and Fig. 1). Compared to the original COMPERA 2.0 risk score, the modified and hybrid versions tended to classify patients into higher grade (Fig. 1, Additional file 3: Tables S3 and S4). Similar results were also observed in the 3-S model (Fig. 1). The incoherence in risk stratification between the original and modified/hybrid versions is presented in Fig. 1 and Additional file 3: Tables S5–S8.

**Table 2:** Baseline characteristics of all included patients, stratified by the original COMPERA 2.0 4-stratum

Variables	All (n = 175)	Low risk (n = 37)	Intermediate-low risk(n = 52)	Intermediate-high risk (n = 74)	High risk (n = 12)	*P*-value
Demographics						
Age, years	60.1 ± 10.8	54.2 ± 12.4	62.4 ± 9.4	61.2 ± 10.2	61.1 ± 10.1	0.003
Body mass index, kg/m^2^	23.9 ± 3.4	25.5 ± 3.5	23.7 ± 2.9	23.7 ± 3.6	21.9 ± 2.3	0.005
Female, n (%)	92 (52.6)	16 (43.2)	32 (61.5)	37 (50)	7 (58.3)	0.344
Disease duration, years	3 (1, 7)	2 (1, 4)	3 (1, 6)	4.0 (2.0, 8.3)	4.5 (2.4, 7.3)	0.025
WHO-FC						< 0.001
I, n (%)	1 (0.6)	1 (2.7)	0	0	0	
II, n (%)	61 (34.9)	36 (97.3)	24 (46.2)	1 (1.4)	0	
III, n (%)	108 (61.7)	0	28 (53.8)	72 (97.3)	8 (66.7)	
IV, n (%)	5 (2.9)	0	0	1 (1.4)	4 (33.3)	
NT-proBNP, ng/L	759.9 (209.0, 1659.3)	106.6 (52.5, 223.6)	379.0 (178.0, 974.5)	1491.5 (823.5, 2671.3)	2502.0 (1831.3, 2738.0)	< 0.001
6MWD, m	350.5 ± 110.6	451.7 ± 74.6	375.9 ± 88.6	312.3 ± 84.2	163.0 ± 78.3	< 0.001
PH specific medicine						0.252
None, n (%)	54 (30.9)	16 (43.2)	19 (36.5)	16 (21.6)	3 (25)	
Mono, n (%)	109 (62.3)	18 (48.6)	29 (55.8)	54 (73)	8 (66.7)	
Double, n (%)	12 (6.9)	3 (8.1)	4 (7.7)	4 (5.4)	1 (8.3)	
Echocardiography						
RVED/LVED	0.84 ± 0.25	0.66 ± 0.13	0.73 ± 0.16	0.96 ± 0.26	1.06 ± 0.21	< 0.001
EF, %	65.1 ± 6.2	65.9 ± 5.7	64.8 ± 5.5	64.7 ± 6.6	66.6 ± 8.1	0.664
TRV, m/s	4.28 ± 0.68	3.84 ± 0.85	4.15 ± 0.63	4.53 ± 0.48	4.61 ± 0.63	< 0.001
Hemodynamics						
S_v_O_2_, %	66.8 ± 8.29	71.1 ± 4.7	68.7 ± 10.5	63.9 ± 6.7	62.54 ± 6.8	< 0.001
mRAP, mmHg	8 (5, 9)	7.0 (4.5, 9.0)	7 (5, 8)	8 (6, 10)	11.5 (8.3, 17.0)	0.001
mPAP, mmHg	49.4 ± 11.8	43.1 ± 9.9	45.3 ± 12.6	54.8 ± 9.5	53.2 ± 9.9	< 0.001
PAWP, mmHg	10.4 ± 3.4	10.7 ± 1.6	10.0 ± 2.0	10.2 ± 2.4	11.9 ± 3.1	0.041
CI, L/min/m^2^	2.86 ± 0.72	3.23 ± 0.53	2.89 ± 0.47	2.46 ± 0.54	2.36 ± 0.73	< 0.001
PVR, wood units	10.4 ± 4.6	6.61 ± 2.75	8.74 ± 3.56	13.18 ± 4.09	12.81 ± 4.66	< 0.001

Data are presented as mean ± standard deviation, median (interquartile range) or number (percentage)

*CI* cardiac index, *EF* ejection fraction, *LVED* left ventricular end-diastolic diameter, *mPAP* mean pulmonary artery pressure, *mRAP* mean right atrial pressure, *NT-proBNP* N-terminal pro-brain natriuretic peptide, *PAWP* pulmonary artery wedge pressure, *PVR* pulmonary vascular resistance, *RVED* right ventricular end-diastolic diameter, *6MWD* 6-min walk distance, *S*_*v*_*O*_*2*_ mixed venous oxygen saturation, *TRV* tricuspid regurgitation velocity, *WHO-FC* World Health Organization function class

**Fig. 2:**
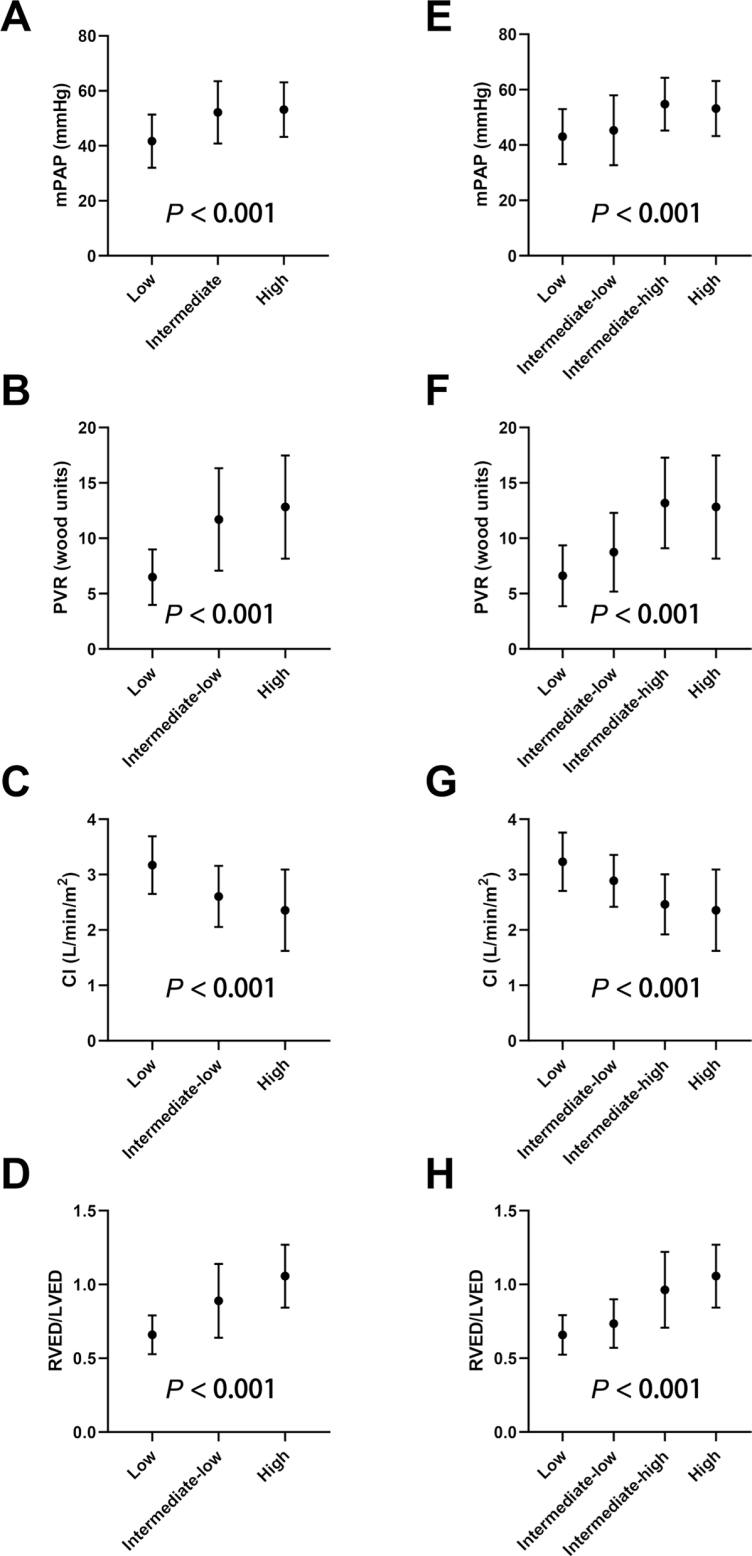
The baseline echocardiographic and hemodynamic characteristics stratified by the original COMERA 2.0 risk score. **A**–**D** The 3-stratum model. **E**–**H** The 4-stratum model

There was a trend that echocardiographic and hemodynamic parameters deteriorated as the risk score increased (Table 2, Additional file 3: Tables S3, S4 and S9–S14, Fig. 2 and Additional file 2: Figures S1, S2). All versions of the COMPERA 2.0 4-S model could further discriminate the differences in echocardiographic and hemodynamic parameters within the intermediate risk group defined by the 3-S model (Fig. 2 and Additional file 2: Figures S1, S2). The median follow-up period was 16 (8, 29) months. During follow-up, 26 (14.9%) of 175 patients experienced clinical worsening: all-cause death (n = 2), unplanned PH-related hospitalization (n = 7), unsatisfactory long-term clinical response (n = 6), and appearance or worsening of signs/symptoms of right heart failure (n = 11).

**Fig. 3:**
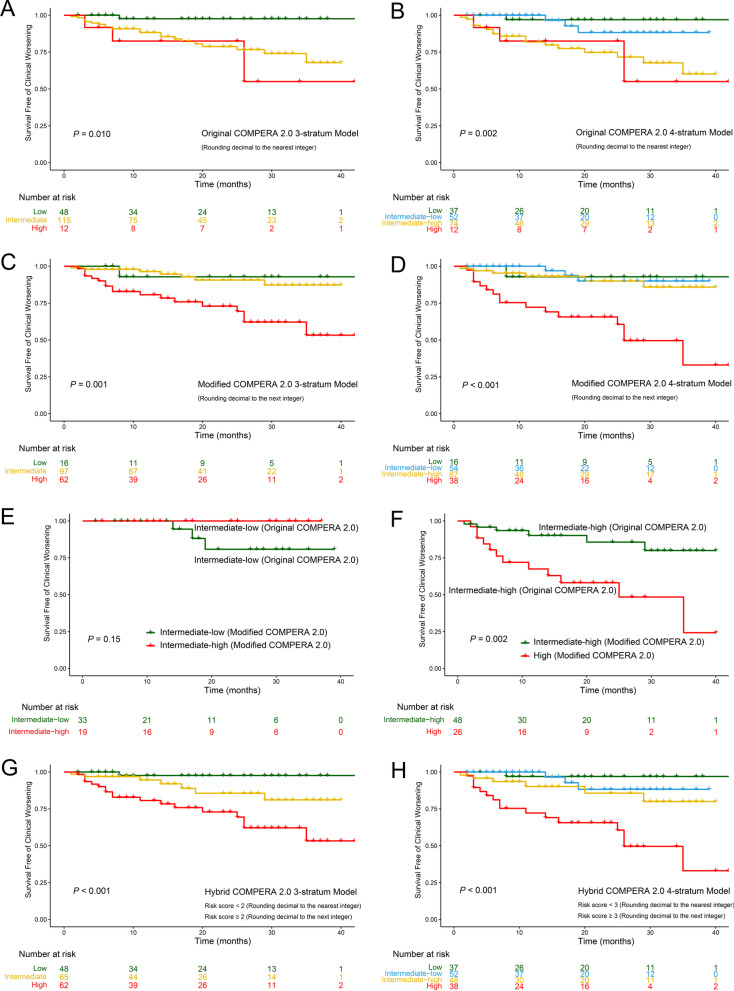
Kaplan–Meier survival curves based on the baseline risk score. **A**, **B** The original COMPERA 2.0 models. **C**, **D** The modified COMPERA 2.0 models. **E**, **F** Using the modified COMPERA 2.0 risk score to further dichotomize patients with intermediate-low and intermediate-high risk in the original version. **G**, **H** The hybrid COMPERA 2.0 models

The 1-, 2- and 3-years clinical worsening-free survival rates after the first BPA session for each risk stratum are summarized in Additional file 3: Table S15 and Fig. 3. Briefly, the cumulative clinical worsening-free survival rates decreased as the baseline risk score escalated. As shown in Fig. 3A and B, the original COMPERA 4-S model could further discriminated patients with intermediate-low risk from those with intermediate risk defined by the 3-S model. Figure 3C and D showed that the modified COMPERA 2.0 risk score seemed to be good at identifying patients at high risk for clinical worsening. We further used the modified COMPERA 2.0 risk score to dichotomize patients with intermediate-low and intermediate-high risk in the original version (Fig. 3E and F). The results showed that the modified COMPERA 2.0 risk score could further divide intermediate-high group defined by original version into two groups (Fig. 3F). The hybrid version seemed to inherit the merits of both original and modified versions (Fig. 3G and H). The Harrell’s C-index of these prediction models decreased in the following order: 0.767 (the hybrid 4-S model) > 0.737 (the modified 4-S model) > 0.714 (the hybrid 3-S model) > 0.707 (the original 4-S model) > 0.685 (the modified 3-S model) > 0.640 (the original 3-S model). The Harrell’s C-index of COMPERA 2.0 prediction models in the present study and published articles are summarized in Table 3. Given the hybrid version showed superiority over original and modified versions in predicting clinical worsening, we would only compare the original 4-S model to the hybrid one.

**Table 3:** The discriminatory power (Harrell’s C-index) of the COMPERA 2.0 risk score in the present study and published articles

	Patients (n)	Outcome	Harrell’s C-index at baseline		Harrell’s C-index at follow-up	
			3-stratum	4-stratum	3-stratum	4-stratum
Hoeper et al. [7]	PAH (1655)	Mortality	Not reported			
Boucly et al. [8]	PAH (2879)	Mortality	0.63	0.67	0.69	0.73
Stubbs et al. [9]	Medically-treated CTEPH (128)	Mortality	0.62	0.62	0.65	0.70
The present study (The original version)	BPA-treated CTEPH (175)	Clinical worsening	0.640	0.707	0.680	0.752
The present study (The modified version)	BPA-treated CTEPH (175)	Clinical worsening	0.685	0.737	0.624	0.724
The present study (The hybrid version)	BPA-treated CTEPH (175)	Clinical worsening	0.714	0.767	0.705	0.748

*PAH* pulmonary arterial hypertension, *CTEPH* chronic thromboembolic pulmonary hypertension

Univariable Cox analysis showed that both the original and hybrid 4-S models were associated with clinical worsening (Table 4). After adjusting for confounders, both the original and hybrid 4-S models could still predict clinical worsening in BPA-treated patients with CTEPH (Additional file 3: Tables S16, S17).

**Table 4:** Associations between the original/hybrid 4-stratum model and clinical outcome

Outcome	No. of patients	No. of events	Crude HR^d^ (95% CI)	*P* for trend
Primary endpoint-clinical worsening				
Original 4-stratum model before the 1st BPA				
Low	37	1	1 (reference)	< 0.001
Intermediate-low	52	3	2.411 (0.251–23.197)	
Intermediate-high	74	18	10.579 (1.411–79.288)	
High	12	4	12.524 (1.397–112.234)	
Original 4-stratum model after the 1st BPA				
Low	77	4	1 (reference)	< 0.001
Intermediate-low	70	10	3.376 (1.057–10.777)	
Intermediate-high	25	12	14.716 (4.709–45.989)	
High	3	0	NA^a^	
Hybrid 4-stratum model before the 1st BPA				
Low	37	1	1 (reference)	< 0.001
Intermediate-low	52	3	2.418 (0.251–23.264)	
Intermediate-high	38	6	5.162 (0.612–42.911)	
High	48	16	18.661 (2.471–140.916)	
Hybrid 4-stratum model after the 1st BPA				
Low	77	4	1 (reference)	< 0.001
Intermediate-low	70	10	3.381 (1.059–10.795)	
Intermediate-high	20	9	15.346 (4.684–50.283)	
High	8	3	9.113 (2.021–41.100)	
Secondary endpoint-achieving low risk^b^				
Original 4-stratum model before the 1st BPA				
Intermediate-low	52	43	1 (reference)	< 0.001
Intermediate-high	74	37	0.209 (0.089–0.490)	
High	12	4	0.105 (0.026–0.424)	
Hybrid 4-stratum model before the 1st BPA				
Intermediate-low	52	43	1 (reference)	< 0.001
Intermediate-high	48	31	0.382 (0.150–0.968)	
High	38	10	0.075 (0.027–0.207)	
Secondary endpoint-achieving mPAP < 30 mmHg^c^				
Original 4-stratum model before the 1st BPA				
Low	23	12	1 (reference)	< 0.001
Intermediate-low	33	10	0.399 (0.132–1.203)	
Intermediate-high	56	9	0.176 (0.059–0.520)	
High	7	0	NA^a^	
Hybrid 4-stratum model before the 1st BPA				
Low	23	12	1 (reference)	< 0.001
Intermediate-low	33	10	0.399 (0.132–1.203)	
Intermediate-high	42	8	0.216 (0.070–0.663)	
High	21	1	0.046 (0.005–0.401)	

*BPA* balloon pulmonary angioplasty, *mPAP* mean pulmonary arterial pressure

^a^No event happened and HR/OR could not be calculated

^b^Only patients with intermediate-low risk or higher grade at baseline were included in the analysis

^c^Only patients with mPAP ≥ 30 mmHg at baseline were included in the analysis

^d^Results of multivariable regression were provided in Additional file 2

### Risk stratification after the first BPA session and clinical worsening

**Fig. 4:**
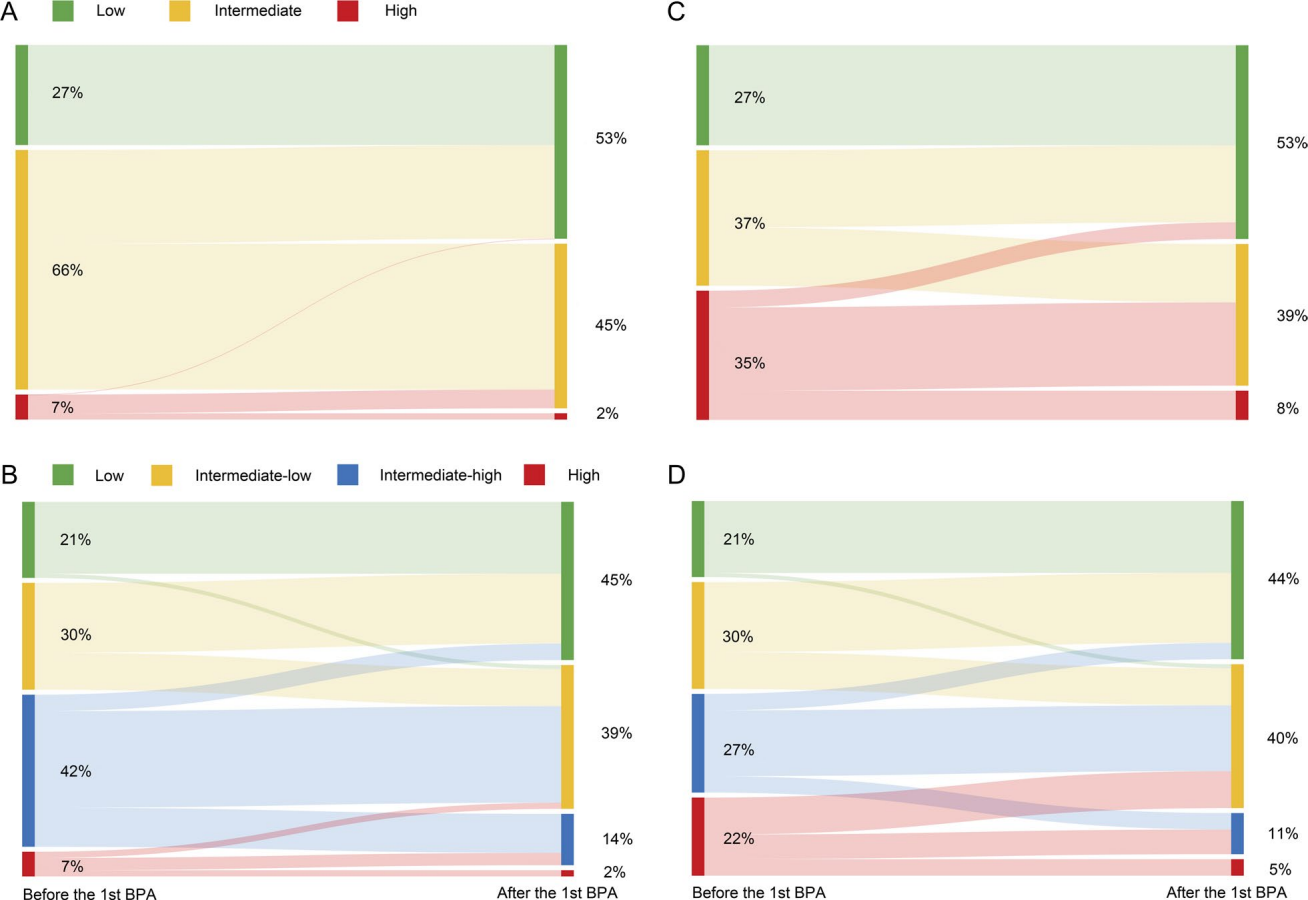
Sankey diagrams showing changes in risk profiles before and after the first BPA using the 3- and 4-stratum method of the original COMPERA 2.0 risk score (**A**, **B**) and the 3- and 4-stratum method of the hybrid COMPERA 2.0 risk score (**C**, **D**). Each panel shows the flow of patients between risk strata (nodes) from baseline to the first re-assessment after the first BPA. The width of each band is weighted to the proportion of patients who had a given trajectory

After the first BPA procedure, 98 (56%) patients improved their risk score in the original 4-S model in comparison to 54 (30.9%) in the original 3-S model (Additional file 3: Tables S18 and S19, Fig. 4). Similarly, 104 (59.4%) patients improved their risk score in the hybrid 4-S model in comparison to 85 (48.6%) in the hybrid 3-S model (Additional file 3: Tables S20, S21, Fig. 4).

**Fig. 5:**
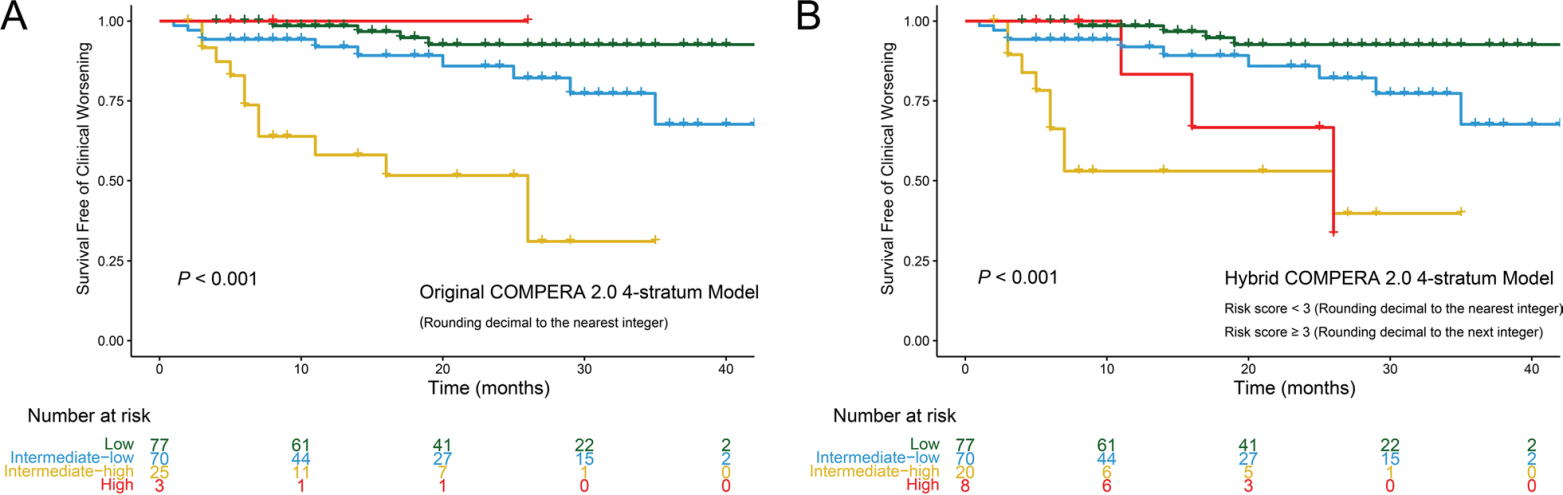
Kaplan–Meier survival curves based on the risk score within 7 days after the first BPA session. **A** The original COMPERA 2.0 4-statum model. The reason for the best clinical-worsening free survival in high-risk group should be attributed to its small sample size. **B** The modified COMPERA 2.0 4-statum model

Like the situation at baseline, the cumulative clinical worsening-free survival rates decreased as the risk score after the first BPA escalated (Fig. 5). The Harrell’s C-index for original and hybrid 4-S risk score in predicting clinical worsening was 0.752 and 0.748 (Table 3), respectively. After adjusting confounders in multivariable Cox analysis, in both original and hybrid version, risk stratification after the first BPA could still predict clinical worsening in BPA-treated patients with CTEPH (Table 4 and Additional file 3: Tables S16, S17).

**Fig. 6:**
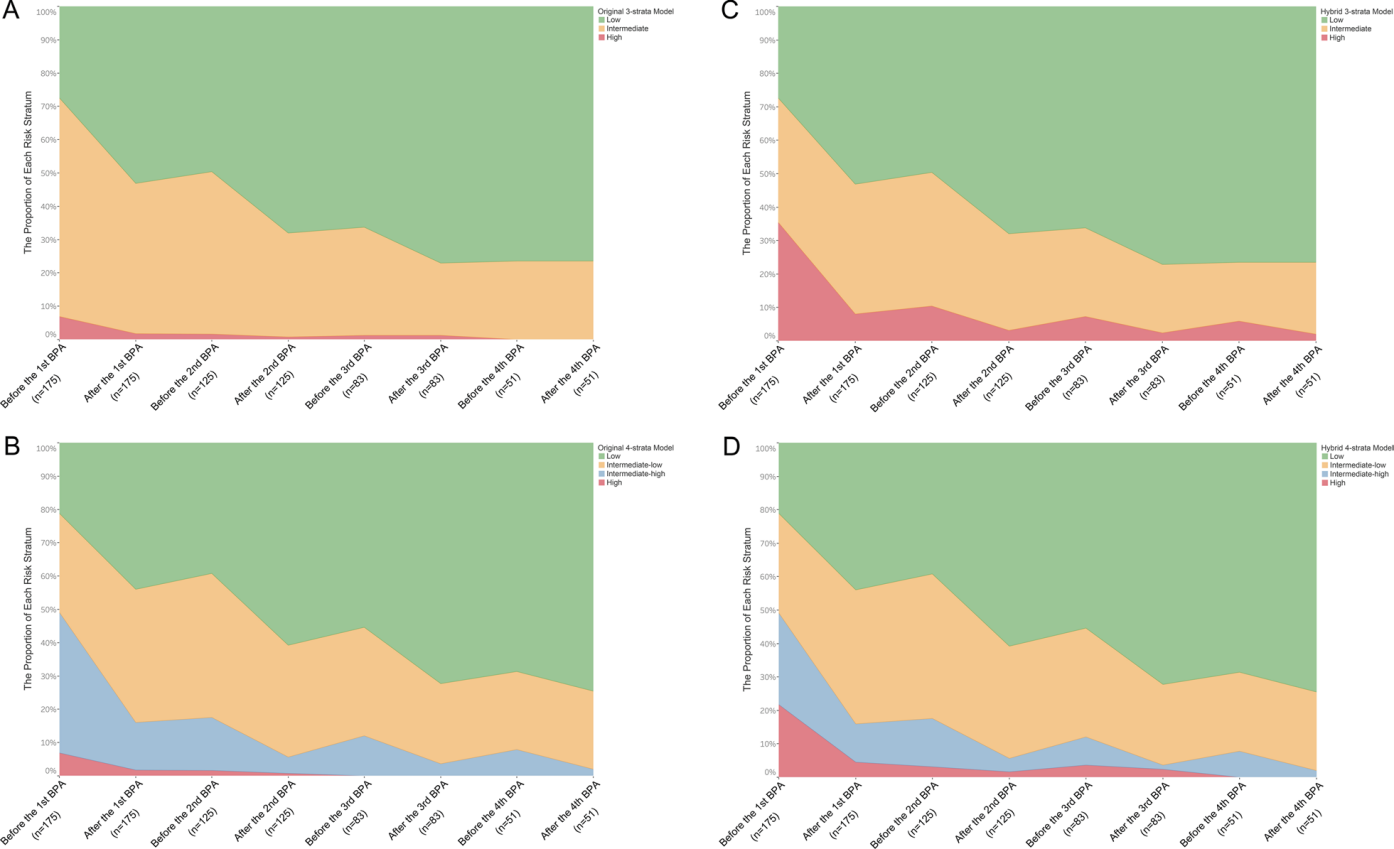
The changes in risk profiles at the procedural level. **A**, **B** the 3- and 4-stratum methods of the original COMPERA 2.0 risk score. **C**, **D** the 3- and 4-stratum methods of the hybrid COMPERA 2.0 risk score

During the subsequent BPA sessions, high risk group defined by the original 4-S model was eliminated at the preoperative evaluation of the third BPA session, while it was at the preoperative evaluation of the fourth BPA session in the original 3-stata model (Fig. 6A and B). By contrast, high risk group defined by the hybrid 4-S model was eliminated at the preoperative evaluation of the fourth BPA session, while the high-risk group still existed after the fourth BPA session in the hybrid 3-stata model (Fig. 6C and D).

### Change in risk stratification from baseline to after the first BPA session and clinical worsening

We performed a subgroup analysis in patients with intermediate-high and high risk at baseline. Those who were improved to intermediate-low or low risk after the first BPA session were less likely to clinically deteriorate compared to those who remained intermediate-high or high risk (the original and hybrid 4-S model were the same: HR = 0.278, 95CI% 0.118–0.654, *P* = 0.003).

### Secondary clinical outcomes

#### Achieving low risk

**Fig. 7:**
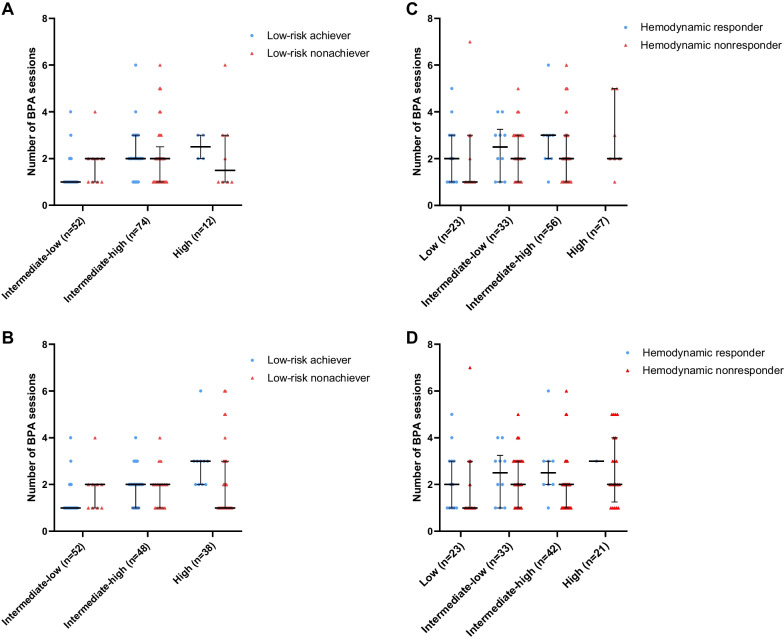
The correlation between the number of BPA session and the occurrence of secondary endpoints, stratified by the risk profiles at baseline. **A**, **C** The original 4- stratum model. **B**, **D** The hybrid 4-stratum model. Data are presented as median (interquartile range)

As illustrated in the “Materials and methods” section, the original and hybrid COMPERA 4-S models shared a same criterion of low risk (Table 1) and the number of patients with intermediate-low risk or higher grade were the same in these two models. When determining the prognostic value of the original and hybrid COMPERA 4-S models in predicting low risk, only 138 patients with intermediate-low risk or higher grade at baseline were included in the analysis. Among those 138 patients, 84 (60.9%) patients achieved low risk after the first BPA session or during subsequent BPA sessions. Figure 7A and B shows that the number of BPA sessions required to achieve low risk increased as the baseline risk score escalated.

Using the original 4-S model, the percentage of achieving low risk at follow-up was 82.7% in the intermediate-low risk group, 50% in the intermediate-high risk group, and 33.3% in the high-risk group. Using the hybrid 4-S model, the percentage of achieving low risk at follow-up was 82.7% in the intermediate-low risk group, 64.6% in the intermediate-high risk group, and 26.3% in the high-risk group.

The area under the curve of original and hybrid 4-S model in predicting achieving low risk was 0.689 (95% CI 0.600–0.778) and 0.750 (95% CI 0.664–0.835), respectively. After adjusting for confounders in multivariable logistic analysis, the original and hybrid 4-S model could still predict achieving low risk after the first BPA session or during the subsequent sessions (Table 4 and Additional file 3: Tables S22, S23).

We performed a subgroup analysis in patients with intermediate-high and high risk at baseline. Those who were improved to intermediate-low risk after the first BPA session were more likely to achieve low risk after the first BPA session or during the subsequent sessions, compared to those who remained intermediate-high or high risk (the original and hybrid 4-S models were the same: OR = 11.207, 95CI% 3.084–40.728, *P* < 0.001).

#### Positive hemodynamic response

Among included 175 patients, 125 patients returned for the assessment of further BPA sessions and underwent reevaluation right heart catheterization. When determining the prognostic value of the original and hybrid 4-S models in predicting positive hemodynamic response, only 119 of 125 patients with mPAP ≥ 30 mmHg at baseline were included in the analysis. Among those 119 patients, 31 (26.1%) patients reached mPAP < 30 mmHg during subsequent BPA sessions. Figure 7C and D shows that the number of BPA sessions required to achieve mPAP < 30 mmHg increased as the baseline risk score escalated.

Using the original 4-S model, the percentage of positive hemodynamic response at follow-up was 52.2% in the low-risk group; 30.3% in the intermediate-low risk group; 16.1% in the intermediate-high risk group; 0% in the high-risk group. Using the hybrid 4-S model, the percentage of positive hemodynamic response at follow-up was 52.2% in the low-risk group; 30.3% in the intermediate-low risk group; 19% in the intermediate-high risk group; 4.8% in the high-risk group.

The area under the curve of original and hybrid 4-S models in predicting achieving mPAP < 30 mmHg was 0.704 (95% CI 0.597–0.810) and 0.715 (95% CI 0.613–0.818), respectively. After adjusting for confounders in multivariable logistic analysis, the original and hybrid 4-S models could still predict achieving mPAP < 30 mmHg at follow-up (Table 4 and Additional file 3: Tables S24, S25).

We performed a subgroup analysis in patients with intermediate-high and high risk at baseline. The percentage of positive hemodynamic response was 19.6% in patients who were improved to intermediate-low or low risk after the first BPA session, while it was 0% in patients remained intermediate-high or high risk. Original and hybrid 4-S model yielded same results. Odds ratio could not be calculated because no outcome happened in patients who remained intermediate-high or high risk.

## Discussion

In a retrospective cohort of BPA-treated patients with CTEPH, we evaluated the prognostic value of the original, modified and hybrid COMPERA 2.0 3-S and 4-S risk score. Our main findings were: (1) All versions of COMPERA 2.0 4-S model outperformed the 3-S one in discriminating the differences in echocardiographic and hemodynamic parameters, and clinical worsening-free survival rates; (2) both the original and hybrid COMPERA 2.0 prediction models could predict clinical outcome, and the hybrid version tended to perform better; (3) the first BPA session could significantly improve the risk profiles; (4) risk stratification within 7 days after the first BPA session could also predict clinical worsening events; (5) the proportion of patients achieving low or intermediate-low risk profiles increased proportionally to the increment of BPA sessions; (6) in patients with intermediate-high or high risk at baseline of the original and hybrid 4-S versions, those who were improved to intermediate-low or low risk after the first BPA session had better clinical outcome than those who remained intermediate-high or high risk; (7) the number of BPA sessions required to achieve low risk/mPAP < 30 mmHg increased as the baseline risk score was higher; (8) low-risk profile was a more easily reachable endpoint than hemodynamic normalization. (9) We proposed to reach a low-risk profile as the first significant endpoint in guiding the BPA procedure following a second hemodynamic endpoint (mPAP < 30 mmHg or < 25 mmHg or even < 20 mmHg) (“two-step endpoint” strategy).

### Three-stratum model vs. four-stratum model

We revealed that all versions of COMPERA 2.0 4-S model could further discriminate the differences in echocardiographic and hemodynamic parameters, and clinical worsening-free survival rates within the intermediate risk group defined by the 3-S model. Moreover, the 4-S model was more sensitive to reflect patients’ response to BPA at a single session level (Additional file 3: Tables S18–S21, Figs. 4 and 6). The current study supports replacing the 3-S model with the 4-S one in BPA-treated patients with CTEPH, which extends the results of COMPERA 2.0 from medically managed CTEPH patients [9].

### Risk stratification and the versions of COMPERA 2.0 4-stratum model

In the present study, approximately 70% of included patients with CTEPH were at intermediate risk in the original COMPERA 2.0 3-S risk score, which was consistent with the previous studies [2, 4]. If we changed the method of stratifying patients from the original COMPERA 2.0 risk score to its two derivative versions (Fig. 1 and Additional file 3: Tables S7, S8), 14.9% and 37.7% of the included patients would have worse risk profiles in the hybrid and modified 4-S model, respectively. The difference in which the decimal number was processed between the original and modified/hybrid COMPERA risk models led to rate patients at higher risk grade.

### Risk stratification at baseline and clinical worsening

In the present study, we found that all 3 versions of COMPERA 2.0 prediction model at baseline were associated with clinical worsening events. Kaplan–Meier curve analysis showed that the original 4-S model could discriminate patients with intermediate-low risk from those with intermediate-high risk, while the modified 4-srata version seemed to be good at identifying patients at high risk for clinical worsening. More importantly, the modified COMPERA 2.0 prediction model could further divide intermediate-high group defined by the original version into two groups (Fig. 3F). The hybrid 4-S version had the highest Harrell’s C-index (original version: 0.707; modified version: 0.737; hybrid version: 0.767), which could be secondary to the fusion of both versions (original and modified). Although differences between studies should be interpreted with caution, Harrell’s C-index of the hybrid COMPERA 2.0 4-S model seemed to be higher than that of the original 4-S one in the published papers (Table 3). To the best of our knowledge, this is the first study to report the prognostic value of the COMPERA 2.0 prediction model in BPA-treated patients with CTEPH.

To reduce the risk of complications, BPA is usually performed in a staged approach with a limited number of pulmonary vessels treated during each session. Approximately 3–10 sessions are required to achieve desirable hemodynamic results [14, 15]. The time interval between 2 sessions has been reported to range from 1 to 8 weeks [16–19], and it might take more than 1 year from the first to the final session [20]. Therefore, the original and hybrid COMPERA 2.0 risk score might be a useful tool that could predict the clinical outcome prior to the beginning of this lengthy process.

### Risk stratification after the first BPA session and clinical worsening

Our results showed that the first BPA session could significantly improve patients’ risk profiles. The 4-S model could more sensitively reflect the change of patients’ clinical status, compared to 3-S one (Fig. 4). Moreover, risk score within 7 days after the first BPA, in both original and hybrid versions, could still predict clinical worsening in BPA-treated patients with CTEPH.

Additionally, the proportion of patients reached low and intermediate-low risk profiles continued to increase during the subsequent BPA sessions. Patients with high risk profile were eliminated at the preoperative evaluation of the third BPA session in the original 4-S model, and of the fourth BPA session in the hybrid version (Fig. 6).

### Change in risk stratification from baseline to after first BPA session and clinical worsening

Subgroup analysis showed that, in patients with intermediate-high or high risk profile at baseline, those who were improved to intermediate-low or low risk after the first BPA session had better clinical worsening-free survival rates, compared to those who remained intermediate-high or high risk. This encourages the clinicians to repeatedly calculate original/hybrid COMPERA 2.0 risk score throughout the whole process to better monitor patients’ response to BPA.

### Secondary clinical outcomes

Our results showed that both the original and hybrid COMPERA 2.0 risk score could predict achieve low risk and mPAP < 30 mmHg at follow-up, and the hybrid version tended to have higher predictive power. Like the primary endpoint, patients who were improved to intermediate-low or low risk after the first BPA session were more likely to achieve secondary endpoints, compared to those who remained intermediate-high or high risk.

### The proposed “two-step endpoint” BPA strategy

There is no consensus on the criterion to finish the BPA procedure. Hemodynamic endpoints like mPAP < 30 mmHg or < 25 mmHg have been used in the published articles, which could take more than 1 year to reach [21–26]. Moreover, it has been reported that BPA could also improve 6MWD, NT-proBNP, and hemodynamics in patients with mPAP < 25 mmHg [27].

In the present study, 60.9% of patients with intermediate-low or higher risk profiles at baseline reached low risk, while only 26.1% of patients with mPAP ≥ 30 mmHg at baseline reached mPAP < 30 mmHg during the follow-up. Hence, we proposed a “two-step endpoint” BPA strategy, in which the first significant endpoint is to reach a low-risk profile (like the recommended treatment goal in pulmonary arterial hypertension), and the second step is to achieve the hemodynamic endpoint (mPAP < 30 mmHg or < 25 mmHg or even < 20 mmHg).

## Study limitations

Our study has several limitations. First, the number of included patients was relatively small (n = 175), resulting in an even smaller population in each risk stratum in comparison to the original study developing the COMPERA 2.0 risk score that enrolled 1655 patients [7]. This might explain why the performance of the original and modified 4-S models was less satisfying in the present study, because a certain number of outcomes are required to discriminate different groups. Also, relatively small sample size prevented us from performing a more comprehensive subgroup analysis such as pairwise comparisons in Kaplan–Meier survival curves. However, it should be emphasized that in published articles regarding BPA, the single-centered sample size of patients was also relatively small, ranging from 8 to 308 [14, 15]. Second, the number of BPA session was relatively small in the present study [median BPA sessions: 3 (2, 4)/per person], while the mean BPA session in the published articles ranged from 1 to 6/per person [14, 15]. Hemodynamic nonresponders and low risk nonachievers in the present study may convert in the future BPA sessions. Our results should be interpreted as patients with lower risk profiles had a better chance to achieve low risk profile/hemodynamic endpoints with less BPA sessions and medical costs required, compared to those with higher risk profiles.

## Conclusion

The COMPERA 2.0 4-S model outperformed the 3-S one in BPA-treated patients with CTEPH. The 4-S model, especially its hybrid version, could be used to predict clinical outcome before the initiation of BPA and monitor treatment response. Low risk profile might be a clinically significant endpoint in guiding the BPA procedure.

## Supplementary Information

**Additional file 1.** RHC and BPA procedure.

**Additional file 2:** **Figure S1.** The baseline echocardiographic and hemodynamic characteristics stratified by the modified COMERA 2.0 risk score. **Figure S2.** The baseline echocardiographic and hemodynamic characteristics stratified by the hybrid COMERA 2.0 risk score.

**Additional file 3: Table S1.** The Scoring of the COMPERA 2.0 3-stratum Prediction Model. **Table S2.** The Scoring of the COMPERA 2.0 4-stratum Prediction Model. **Table S3.** Baseline characteristics of all included patients, stratified by the modified COMPERA 2.0 4-stratum. **Table S4.** Baseline characteristics of all included patients, stratified by the hybrid COMPERA 2.0 4-stratum. **Table S5.** The incoherence in the risk stratification between the original and modified 3-stratum model. **Table S6.** The incoherence in the risk stratification between the original and hybrid 3-stratum model. **Table S7.** The incoherence in risk stratification between the original and modified 4-stratum model. **Table S8.** The incoherence in risk stratification between the original and hybrid 4-stratum model. **Table S9.** Correlation between the original COMPERA 4-stratum model and echocardiographic/hemodynamic parameters. **Table S10.** Correlation between the original COMPERA 3-stratum model and echocardiographic/hemodynamic parameters. **Table S11.** Correlation between the modified COMPERA 4-stratum model and echocardiographic/hemodynamic parameters. **Table S12.** Correlation between the modified COMPERA 3-stratum model and echocardiographic/hemodynamic parameters. **Table S13.** Correlation between the hybrid COMPERA 4-stratum model and echocardiographic/hemodynamic parameters. **Table S14.** Correlation between the hybrid COMPERA 3-stratum model and echocardiographic/hemodynamic parameters. **Table S15.** The 1-, 2- and 3-year cumulative clinical worsening-free survival rates after the first BPA session for each stratum. **Table S16.** Univariate cox analysis of predictive variables of clinical worsening. **Table S17.** Multivariate cox analysis of predictive variables of clinical worsening. **Table S18.** Change in risk stratum after the 1st BPA session by risk at baseline (the original 3-stratum model). **Table S19.** Change in Risk stratum after the 1st BPA session by risk at baseline (original 4-stratum model). **Table S20.** Change in risk stratum after the 1st BPA session by risk at baseline (the hybrid 3-stratum model). **Table S21.** Change in risk stratum after the 1st BPA session by risk at baseline with (hybrid 4-stratum model). **Table S22.** Univariate logistic analysis of variables in predicating reaching low risk after the first BPA session or during subsequent sessions. **Table S23.** Multivariate logistic analysis of variables in predicating reaching low risk after the first BPA session or during subsequent sessions. **Table S24.** Univariate logistic analysis of variables in predicating hemodynamic response during subsequent BPA sessions. **Table S25.** Multivariate logistic analysis of variables in predicating hemodynamic response at follow-up.

## Data Availability

The datasets used and/or analysed during the current study are available from the corresponding author on reasonable request.

## Abbreviations

BPA: Balloon pulmonary angioplasty
CTEPH: Chronic thromboembolic pulmonary hypertension
LVED: Left ventricular end-diastolic diameter
mPAP: Mean pulmonary arterial pressure
NT-proBNP: N-terminal pro-brain natriuretic peptide
RHC: Right heart catheterization
RVED: Right ventricular end-diastolic diameter
SPAHR: The Swedish Pulmonary Arterial Hypertension Registry
S_v_O_2_: Mixed venous oxygen saturation
6MWD: 6 Min walk distance
TRV: Tricuspid regurgitation velocity
WHO-FC: World Health Organization functional class
