# Supplementary material for: The original and two new derivative versions of the COMPERA 2.0 risk assessment model: useful tools for guiding balloon pulmonary angioplasty
Source: Respir Res. 2022 Nov 15;23:312. doi: 10.1186/s12931-022-02232-1 (PMC9664665; doi:10.1186/s12931-022-02232-1)
Supplement: Supplementary file 1 — Additional file 1. RHC and BPA procedure. [file 12931_2022_2232_MOESM1_ESM.docx]

***RHC and BPA procedure***

RHC and BPA were routinely performed via the right femoral vein, under local anesthesia, by two interventional cardiologists. RHC was firstly performed prior to each BPA session to obtain the hemodynamics parameters, including right atrial pressure, right ventricular pressure, systolic-, diastolic- and mean PAP, PAWP, cardiac output (calculated by indirect Fick’s principle) and oxygen saturation (vena cava, right atrium, right ventricle, and pulmonary artery). S_v_O_2_, CI and PVR were calculated according to standard formulas.

After RHC, we performed pulmonary angiography, in anterior-posterior and lateral (60 degree) projections, to acquire an overall view of the filling defect. Subsequently, a 70 cm 6F-7F long sheath (Flexor® Check-Flo® Introducer; Cook Medical, Bloomington, IN, USA), via the right femoral vein, was inserted into the lobar pulmonary artery to introduce a 6F guiding catheter in order to prevent the guiding catheter from moving along with the beating heart as well as to facilitate the exchange of guiding catheter, wires and balloons. 6 Fr Multi-purpose (Cordis Corporation, Bridgewater, New Jersey, USA), Amplatz Left (mainly for anterior segments, especially A3; Terumo® Heartrail™ II; Terumo Corporation, Tokyo, Japan) or Judkins Right (Terumo® Heartrail™ II; Terumo Corporation, Tokyo, Japan) was selected as the guiding catheter. After introducing the guiding catheter, 2000 U unfractionated heparin was additionally infused, and oxygen was given at a flow rate of 5–8 L/min to all patients. Priority selection of target lesions was as follows: right lung > left lung, inferior lobe > superior or middle lobes, webs or bands > subtotal occlusion > chronic total occlusion > tortuous lesions. Based on selective pulmonary angiography, a 0.014-in. wire (Hi-Torque Pilot 50; Abbot, Santa Clara, CA, USA) was crossed to the target lesion, then appropriate balloon catheters (Mini Trek; Abbot, Santa Clara, CA, USA) were positioned over the selected lesion and inflated with iodinated contrast agents to pressures of 2–14 atm for 5–30 s. A small-sized balloon (2.0 × 20 mm) or smaller one was firstly used at the initial dilation. Inflation pressure was dynamically adjusted according to balloon size and vessel size. Selective angiography with 1:1 mixture of saline and contrast agents was performed with deep breath holds in real time to confirm vascular filling and the presence of ruptured vessels. Dilations were repeated with unchanged or stepwise increased balloon size in case of unresponsive or poorly responsive target angiographic vessel. Hemodynamic parameters were once again measured by RHC at the end of each BPA session. A typical BPA session lasted for 1–2 h. Each session should not exceed 2000 mGy radiation exposure, 60 min fluoroscopy time, and 250 ml contrast medium.

**References**

Jin Q, Luo Q, Yang T, Zeng Q, Yu X, Yan L, Zhang Y, Zhao Q, Ma X, An C, Xiong C, Zhao Z, Liu Z. Improved hemodynamics and cardiopulmonary function in patients with inoperable chronic thromboembolic pulmonary hypertension after balloon pulmonary angioplasty. Respir Res. 2019;20(1):250. DOI: 10.1186/s12931-019-1211-y.

Jin Q, Zhao ZH, Luo Q, Zhao Q, Yan L, Zhang Y, Li X, Yang T, Zeng QX, Xiong CM, Liu ZH. Balloon pulmonary angioplasty for chronic thromboembolic pulmonary hypertension: State of the art. World J Clin Cases. 2020;8(13):2679-2702. DOI: 10.12998/wjcc.v8.i13.2679.

**Abbreviation:**

BPA, balloon pulmonary angioplasty; CI, cardiac index; mPAP, mean pulmonary artery pressure; PAWP, pulmonary artery wedge pressure; PVR, pulmonary vascular resistance; RHC, right heart catheterization; S_v_O_2_, mixed venous oxygen saturation.
