# Supplementary material for: The original and two new derivative versions of the COMPERA 2.0 risk assessment model: useful tools for guiding balloon pulmonary angioplasty
Source: Respir Res. 2022 Nov 15;23:312. doi: 10.1186/s12931-022-02232-1 (PMC9664665; doi:10.1186/s12931-022-02232-1)
Supplement: Supplementary file 2 — Additional file 2: Figure S1. The baseline echocardiographic and hemodynamic characteristics stratified by the modified COMERA 2.0 risk score. Figure S2. The baseline echocardiographic and hemodynamic characteristics stratified by the hybrid COMERA 2.0 risk score. [file 12931_2022_2232_MOESM2_ESM.docx]

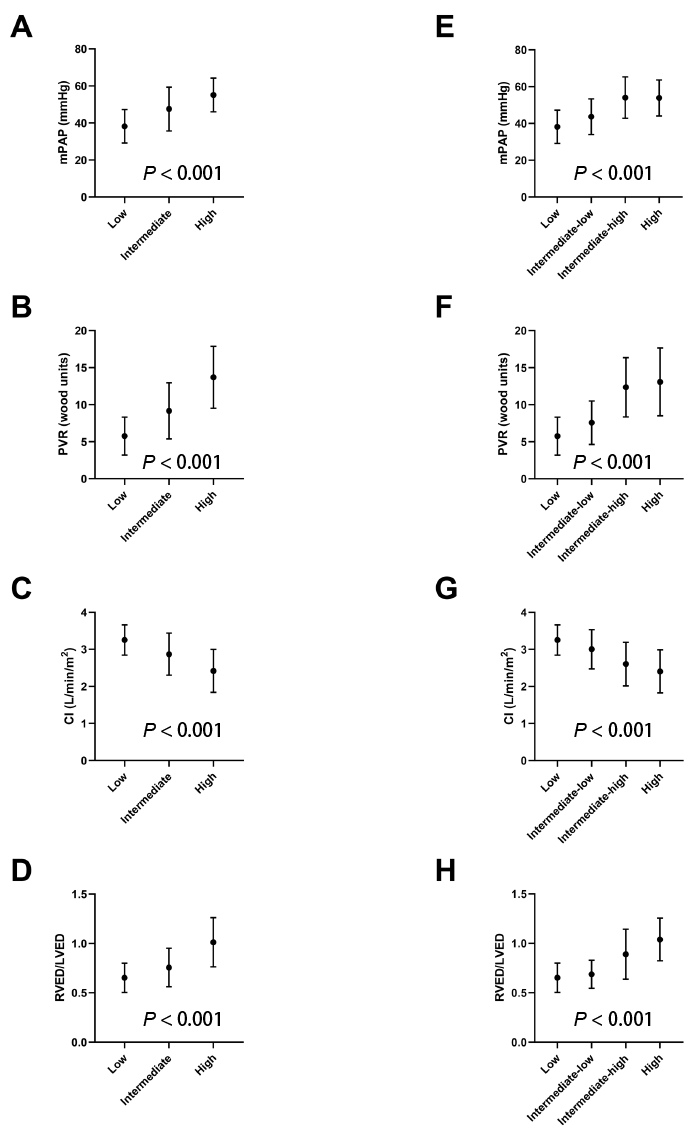


Figure S1. The baseline echocardiographic and hemodynamic characteristics stratified by the modified COMERA 2.0 risk score. (A-D) the 3-stratum model. (E-H) the 4-stratum model. Data are presented as mean ± standard deviation.


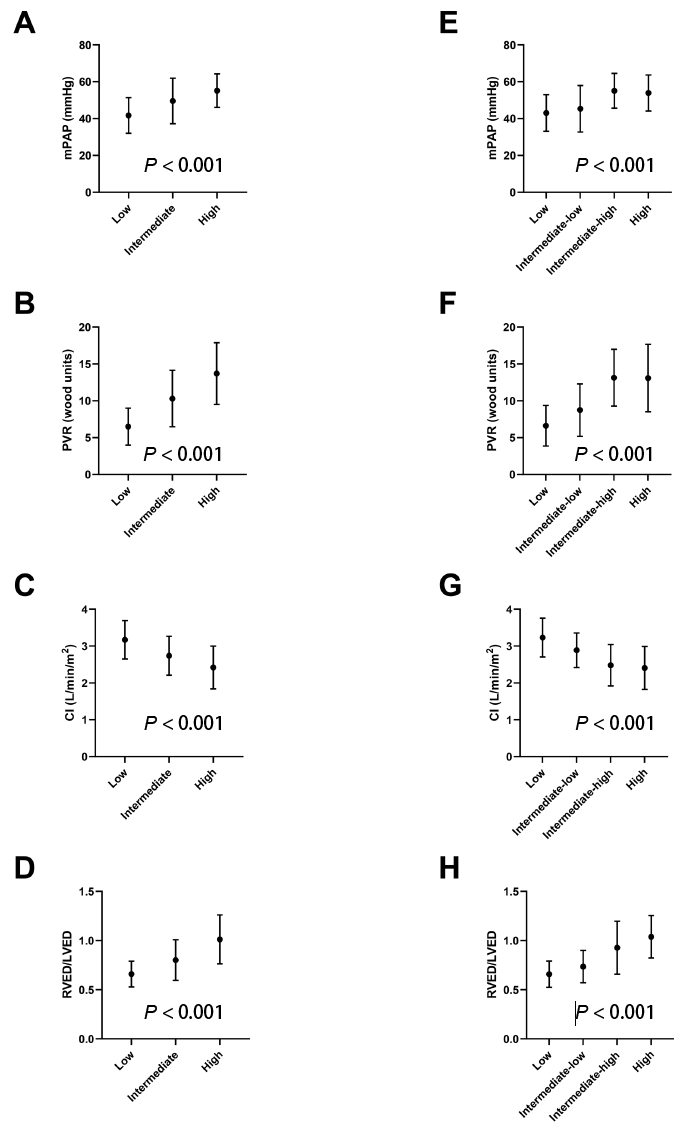


Figure S2. The baseline echocardiographic and hemodynamic characteristics stratified by the hybrid COMERA 2.0 risk score. (A-D) the 3-stratum model. (E-H) the 4-stratum model. Data are presented as mean ± standard deviation
