# Supplementary material for: The original and two new derivative versions of the COMPERA 2.0 risk assessment model: useful tools for guiding balloon pulmonary angioplasty
Source: Respir Res. 2022 Nov 15;23:312. doi: 10.1186/s12931-022-02232-1 (PMC9664665; doi:10.1186/s12931-022-02232-1)
Supplement: Supplementary file 3 — Additional file 3: Table S1. The Scoring of the COMPERA 2.0 3-stratum Prediction Model. Table S2. The Scoring of the COMPERA 2.0 4-stratum Prediction Model. Table S3. Baseline characteristics of all included patients, stratified by the modified COMPERA 2.0 4-stratum. Table S4. Baseline characteristics of all included patients, stratified by the hybrid COMPERA 2.0 4-stratum. Table S5. The incoherence in the risk stratification between the original and modified 3-stratum model. Table S6. The incoherence in the risk stratification between the original and hybrid 3-stratum model. Table S7. The incoherence in risk stratification between the original and modified 4-stratum model. Table S8. The incoherence in risk stratification between the original and hybrid 4-stratum model. Table S9. Correlation between the original COMPERA 4-stratum model and echocardiographic/hemodynamic parameters. Table S10. Correlation between the original COMPERA 3-stratum model and echocardiographic/hemodynamic parameters. Table S11. Correlation between the modified COMPERA 4-stratum model and echocardiographic/hemodynamic parameters. Table S12. Correlation between the modified COMPERA 3-stratum model and echocardiographic/hemodynamic parameters. Table S13. Correlation between the hybrid COMPERA 4-stratum model and echocardiographic/hemodynamic parameters. Table S14. Correlation between the hybrid COMPERA 3-stratum model and echocardiographic/hemodynamic parameters. Table S15. The 1-, 2- and 3-year cumulative clinical worsening-free survival rates after the first BPA session for each stratum. Table S16. Univariate cox analysis of predictive variables of clinical worsening. Table S17. Multivariate cox analysis of predictive variables of clinical worsening. Table S18. Change in risk stratum after the 1st BPA session by risk at baseline (the original 3-stratum model). Table S19. Change in Risk stratum after the 1st BPA session by risk at baseline (original 4-stratum model). Table S20. Change in risk [file 12931_2022_2232_MOESM3_ESM.docx]

Table S1 The Scoring of the COMPERA 2.0 3-stratum Prediction Model

| Points assigned | 1 | 2 | 3 |
| --- | --- | --- | --- |
| WHO-FC | I/II | III | IV |
| 6MWD | >440 m | 440-165 m | <165 m |
| NT-proBNP  Or  BNP | <300 ng/L  <50 ng/L | 300-1100 ng/L  <50-800 ng/L | >1100 ng/L  >800 ng/L |

BNP, brain natriuretic peptide; NT-proBNP, N-terminal pro-brain natriuretic peptide; 6MWD, 6-minute walk distance; WHO-FC, World Health Organization function class.

Table S2 The Scoring of the COMPERA 2.0 4-stratum Prediction Model

| Points assigned | 1 | 2 | 3 | 4 |
| --- | --- | --- | --- | --- |
| WHO-FC | I/II | - | III | IV |
| 6MWD | >440 m | 440-320 m | 319-165 m | <165 m |
| NT-proBNP  Or  BNP | <300 ng/L  <50 ng/L | 300-649 ng/L  50-199 ng/L | 650-1100 ng/L  200-800 ng/L | >1100 ng/L  >800 ng/L |

BNP, brain natriuretic peptide; NT-proBNP, N-terminal pro-brain natriuretic peptide; 6MWD, 6-minute walk distance; WHO-FC, World Health Organization function class.

Table S3 Baseline Characteristics of All Included Patients, Stratified by the Modified COMPERA 2.0 4-stratum.

| Variables | All  (n=175) | Low risk  (n=16) | Intermediate-low risk  (n=54) | Intermediate-high risk  (n=67) | High risk  (n=38) | *P*-value |
| --- | --- | --- | --- | --- | --- | --- |
| Demographics |  |  |  |  |  |  |
| Age, years | 60.1±10.8 | 52.56±13.0 | 60.4±11.1 | 59.4±10.5 | 63.9±8.1 | 0.005 |
| Body mass index, kg/m^2^ | 23.9±3.4 | 25.6±2.5 | 24.2±3.5 | 24.1±3.6 | 22.6±2.7 | 0.016 |
| Female, n (%) | 92 (52.6) | 6 (37.5) | 30 (55.6) | 34 (50.7) | 22 (57.9) | 0.538 |
| Disease duration, years | 3.00 (1.00, 7.00) | 2.00 (1.00, 4.75) | 3.00 (1.00, 6.00) | 3.00 (1.00, 7.00) | 5.00 (2.00, 8.25) | 0.038 |
| WHO-FC |  |  |  |  |  | <0.001 |
| I, n (%) | 1 (0.6) | 0 | 1 (1.9) | 0 | 0 |  |
| II, n (%) | 61 (34.9) | 16 (100) | 35 (64.8) | 10 (14.9) | 0 |  |
| III, n (%) | 108 (61.7) | 0 | 18 (33.3) | 56 (83.6) | 34 (89.5) |  |
| IV, n (%) | 5 (2.9) | 0 | 0 | 1 (1.5) | 4 (10.5) |  |
| NT-proBNP, ng/L | 759.9 (209.0, 1659.3) | 97.7 (40.0, 225.8) | 193.8 (91.5, 298.1) | 1099.0 (719.0, 1702.0) | 2567.0 (1632.0, 3386.5) | <0.001 |
| 6MWD, m | 350.5±110.6 | 512.5±55.6 | 388.0±80.0 | 355.8±78.3 | 219.5±71.4 | <0.001 |
| PH specific medicine |  |  |  |  |  | 0.361 |
| None, n (%) | 54 (30.9) | 7 (43.8) | 21 (38.9) | 18 (26.9) | 8 (21.1) |  |
| Mono, n (%) | 109 (62.3) | 8 (50) | 28 (51.9) | 45 (67.2) | 28 (73.7) |  |
| Double, n (%) | 12 (6.9) | 1 (6.3) | 5 (9.3) | 4 (6) | 2 (5.3) |  |
| Echocardiography |  |  |  |  |  |  |
| RVED/LVED | 0.84±0.25 | 0.65±0.15 | 0.69±0.14 | 0.89±0.25 | 1.04±0.22 | <0.001 |
| EF, % | 65.1±6.2 | 64.8±5.6 | 65.2±5.6 | 64.6±6.4 | 66.1±6.9 | 0.679 |
| TRV, m/s | 4.28±0.68 | 3.76±0.50 | 3.99±0.79 | 4.50±0.59 | 4.52±0.46 | <0.001 |
| Hemodynamics |  |  |  |  |  |  |
| S_v_O_2_, % | 66.8±8.29 | 70.2±4.8 | 70.9±5.4 | 64.9±9.7 | 62.7±7.1 | <0.001 |
| mRAP, mmHg | 8 (5, 9) | 7.5 (4.0, 8.8) | 7 (5, 9) | 8 (6, 9) | 8.0 (6.8, 12.0) | 0.046 |
| mPAP, mmHg | 49.4±11.8 | 38.2±9.1 | 43.7±9.7 | 54.1±11.2 | 53.9±9.8 | <0.001 |
| PAWP, mmHg | 10.4±3.4 | 10.2±3.3 | 10.4±3.1 | 9.9±3.3 | 11.1±4.1 | 0.440 |
| CI, L/min/m^2^ | 2.86±0.72 | 3.32±0.40 | 3.22±0.73 | 2.65±0.65 | 2.51±0.60 | <0.001 |
| PVR, wood units | 10.4±4.6 | 5.78±2.57 | 7.58±2.93 | 12.36±4.00 | 13.08±4.57 | <0.001 |

Data are presented as mean ± standard deviation, median (interquartile range) or number (percentage). CI, cardiac index; EF, ejection fraction; LA, anteroposterior diameter of left atrium; LVED, left ventricular end-diastolic diameter; mPAP, mean pulmonary artery pressure; mRAP, mean right atrial pressure; NT-proBNP, N-terminal pro-brain natriuretic peptide; PAWP, pulmonary artery wedge pressure; PVR, pulmonary vascular resistance; RVED, right ventricular end-diastolic diameter; 6MWD, 6-minute walk distance; S_v_O_2_, mixed venous oxygen saturation; TRV, tricuspid regurgitation velocity; WHO-FC, World Health Organization function class.

Table S4 Baseline Characteristics of All Included Patients, Stratified by the Hybrid COMPERA 2.0 4-stratum.

| Variables | All  (n=175) | Low risk  (n=37) | Intermediate-low risk  (n=52) | Intermediate-high risk  (n=48) | High risk  (n=38) | *P*-value |
| --- | --- | --- | --- | --- | --- | --- |
| Demographics |  |  |  |  |  |  |
| Age, years | 60.1±10.8 | 54.2±12.4 | 62.4±9.4 | 59.0±11.1 | 63.9±8.1 | <0.001 |
| Body mass index, kg/m^2^ | 23.9±3.4 | 25.5±3.5 | 23.7±2.9 | 24.1±3.9 | 22.6±2.7 | 0.003 |
| Female, n (%) | 92 (52.6) | 16 (43.2) | 32 (61.5) | 22 (45.8) | 22 (57.9) | 0.233 |
| Disease duration, years | 3 (1, 7) | 2 (1, 4) | 3 (1, 6) | 3.5 (1.5, 7.5) | 5 (2, 8) | 0.018 |
| WHO-FC |  |  |  |  |  | <0.001 |
| I, n (%) | 1 (0.6) | 1 (2.7) | 0 | 0 | 0 |  |
| II, n (%) | 61 (34.9) | 36 (97.3) | 24 (46.2) | 1 (2.1) | 0 |  |
| III, n (%) | 108 (61.7) | 0 | 28 (53.8) | 46 (95.8) | 34 (89.5) |  |
| IV, n (%) | 5 (2.9) | 0 | 0 | 1 (2.1) | 4 (10.5) |  |
| NT-proBNP, ng/L | 759.9 (209.0, 1659.3) | 106.6 (52.5, 223.6) | 379.0 (178.0, 974.5) | 1128.6 (745.5, 2025.8) | 2567 (1632, 3386.5) | <0.001 |
| 6MWD, m | 350.5±110.6 | 451.7±74.6 | 375.9±88.6 | 348.5±76.3 | 219.5±71.4 | <0.001 |
| PH specific medicine |  |  |  |  |  | 0.247 |
| None, n (%) | 54 (30.9) | 16 (43.2) | 19 (36.5) | 11 (22.9) | 8 (21.1) |  |
| Mono, n (%) | 109 (62.3) | 18 (48.6) | 29 (55.8) | 34 (70.8) | 28 (73.7) |  |
| Double, n (%) | 12 (6.9) | 3 (8.1) | 4 (7.7) | 3 (6.3) | 2 (5.3) |  |
| Echocardiography |  |  |  |  |  |  |
| RVED/LVED | 0.84±0.25 | 0.66±0.12 | 0.73±0.16 | 0.93±0.27 | 1.04±0.22 | <0.001 |
| EF, % | 65.1±6.2 | 65.9±5.7 | 64.8±5.5 | 64.1±6.7 | 66.1±6.9 | 0.415 |
| TRV, m/s | 4.28±0.68 | 3.84±0.85 | 4.15±0.63 | 4.56±0.53 | 4.52±0.46 | <0.001 |
| Hemodynamics |  |  |  |  |  |  |
| S_v_O_2_, % | 66.8±8.29 | 71.1±4.7 | 68.7±10.5 | 64.5±6.3 | 62.7±7.1 | <0.001 |
| mRAP, mmHg | 8 (5, 9) | 7.0 (4.5, 9.0) | 7 (5, 8) | 8.5 (6.0, 10.0) | 8.0 (6.8, 12.0) | 0.002 |
| mPAP, mmHg | 49.4±11.8 | 43.1±9.9 | 45.3±12.6 | 55.1±9.4 | 53.9±9.8 | <0.001 |
| PAWP, mmHg | 10.4±3.4 | 10.7±1.6 | 10.0±2.0 | 10.2±2.0 | 10.8±3.1 | 0.315 |
| CI, L/min/m^2^ | 2.86±0.72 | 3.23±0.53 | 2.89±0.47 | 2.48±0.56 | 2.41±0.58 | <0.001 |
| PVR, wood units | 10.4±4.6 | 6.61±2.75 | 8.74±3.56 | 13.17±3.82 | 13.08±4.57 | <0.001 |

Data are presented as mean ± standard deviation, median (interquartile range) or number (percentage). CI, cardiac index; EF, ejection fraction; LA, anteroposterior diameter of left atrium; LVED, left ventricular end-diastolic diameter; mPAP, mean pulmonary artery pressure; mRAP, mean right atrial pressure; NT-proBNP, N-terminal pro-brain natriuretic peptide; PAWP, pulmonary artery wedge pressure; PVR, pulmonary vascular resistance; RVED, right ventricular end-diastolic diameter; 6MWD, 6-minute walk distance; S_v_O_2_, mixed venous oxygen saturation; TRV, tricuspid regurgitation velocity; WHO-FC, World Health Organization function class.

Table S5 The Incoherence in the Risk Stratification Between the Original and Modified 3-stratum Model

| Risk | Low in the modified model | Intermediate in the modified model | High in the modified model | Sum |
| --- | --- | --- | --- | --- |
| Low in the original model | 16 | 32 | 0 | 48 |
| Intermediate in the original model | 0 | 65 | 50 | 115 |
| High in the original model | 0 | 0 | 12 | 12 |
| Sum | 16 | 97 | 62 | 175 |

Table S6 The Incoherence in the Risk Stratification Between the Original and Hybrid 3-stratum Model

| Risk | Low in the hybrid model | Intermediate in the hybrid model | High in the hybrid model | Sum |
| --- | --- | --- | --- | --- |
| Low in the original model | 48 | 0 | 0 | 48 |
| Intermediate in the original model | 0 | 65 | 50 | 115 |
| High in the original model | 0 | 0 | 12 | 12 |
| Sum | 48 | 65 | 62 | 175 |

Table S7 The Incoherence in Risk Stratification Between the Original and Modified 4-stratum Model

| Risk | Low  in modified model | Intermediate-low  in modified model | Intermediate-high  in modified model | High  in the modified model | Sum |
| --- | --- | --- | --- | --- | --- |
| Low in the original model | 16 | 21 | 0 | 0 | 37 |
| Intermediate-low in the original model | 0 | 33 | 19 | 0 | 52 |
| Intermediate-high in the original model | 0 | 0 | 48 | 26 | 74 |
| High in the original model | 0 | 0 | 0 | 12 | 12 |
| Sum | 16 | 54 | 67 | 38 | 175 |

Table S8 The Incoherence in Risk Stratification Between the Original and Hybrid 4-stratum Model

| Risk | Low  in the modified model | Intermediate-low  in the hybrid model | Intermediate-high  in the hybrid model | High  in the hybrid model | Sum |
| --- | --- | --- | --- | --- | --- |
| Low in the original model | 37 | 0 | 0 | 0 | 37 |
| Intermediate-low in the original model | 0 | 52 | 0 | 0 | 52 |
| Intermediate-high in the original model | 0 | 0 | 48 | 26 | 74 |
| High in original model | 0 | 0 | 0 | 12 | 12 |
| Sum | 37 | 52 | 48 | 38 | 175 |

Table S9 Correlation Between the Original COMPERA 4-stratum Model and Echocardiographic/ Hemodynamic Parameters.

| Variables | Spearman Correlation | *P-*value |
| --- | --- | --- |
| Echocardiography |  |  |
| RVED/LVED | 0.605 | <0.001 |
| TRV, m/s | 0.386 | <0.001 |
| Hemodynamics |  |  |
| S_v_O_2_, % | -0.478 | <0.001 |
| mRAP, mmHg | 0.270 | <0.001 |
| mPAP, mmHg | 0.425 | <0.001 |
| CI, L/min/m^2^ | -0.489 | <0.001 |
| PVR, wood units | 0.602 | <0.001 |

CI, cardiac index; LVED, left ventricular end-diastolic diameter; mPAP, mean pulmonary artery pressure; mRAP, mean right atrial pressure; PVR, pulmonary vascular resistance; RVED, right ventricular end-diastolic diameter; S_v_O_2_, mixed venous oxygen saturation; TRV, tricuspid regurgitation velocity.

Table S10 Correlation Between the Original COMPERA 3-stratum Model and Echocardiographic/ Hemodynamic Parameters.

| Variables | Spearman Correlation | *P-*value |
| --- | --- | --- |
| Echocardiography |  |  |
| RVED/LVED | 0.518 | <0.001 |
| TRV, m/s | 0.385 | <0.001 |
| Hemodynamics |  |  |
| S_v_O_2_, % | -0.374 | <0.001 |
| mRAP, mmHg | 0.190 | 0.012 |
| mPAP, mmHg | 0.381 | <0.001 |
| CI, L/min/m^2^ | -0.424 | <0.001 |
| PVR, wood units | 0.549 | <0.001 |

CI, cardiac index; LVED, left ventricular end-diastolic diameter; mPAP, mean pulmonary artery pressure; mRAP, mean right atrial pressure; PVR, pulmonary vascular resistance; RVED, right ventricular end-diastolic diameter; S_v_O_2_, mixed venous oxygen saturation; TRV, tricuspid regurgitation velocity.

Table S11 Correlation Between the Modified COMPERA 4-stratum Model and Echocardiographic/ Hemodynamic Parameters.

| Variables | Spearman Correlation | *P-*value |
| --- | --- | --- |
| Echocardiography |  |  |
| RVED/LVED | 0.621 | <0.001 |
| TRV, m/s | 0.386 | <0.001 |
| Hemodynamics |  |  |
| S_v_O_2_, % | -0.447 | <0.001 |
| mRAP, mmHg | 0.211 | <0.001 |
| mPAP, mmHg | 0.453 | <0.001 |
| CI, L/min/m^2^ | -0.446 | <0.001 |
| PVR, wood units | 0.587 | <0.001 |

CI, cardiac index; LVED, left ventricular end-diastolic diameter; mPAP, mean pulmonary artery pressure; mRAP, mean right atrial pressure; PVR, pulmonary vascular resistance; RVED, right ventricular end-diastolic diameter; S_v_O_2_, mixed venous oxygen saturation; TRV, tricuspid regurgitation velocity.

Table S12 Correlation Between the Modified COMPERA 3-stratum Model and Echocardiographic/ Hemodynamic Parameters.

| Variables | Spearman Correlation | *P-*value |
| --- | --- | --- |
| Echocardiography |  |  |
| RVED/LVED | 0.573 | <0.001 |
| TRV, m/s | 0.363 | <0.001 |
| Hemodynamics |  |  |
| S_v_O_2_, % | -0.383 | <0.001 |
| mRAP, mmHg | 0.199 | 0.008 |
| mPAP, mmHg | 0.442 | <0.001 |
| CI, L/min/m^2^ | -0.426 | <0.001 |
| PVR, wood units | 0.572 | <0.001 |

CI, cardiac index; LVED, left ventricular end-diastolic diameter; mPAP, mean pulmonary artery pressure; mRAP, mean right atrial pressure; PVR, pulmonary vascular resistance; RVED, right ventricular end-diastolic diameter; S_v_O_2_, mixed venous oxygen saturation; TRV, tricuspid regurgitation velocity.

Table S13 Correlation Between the Hybrid COMPERA 4-stratum Model and Echocardiographic/ Hemodynamic Parameters.

| Variables | Spearman Correlation | *P-*value |
| --- | --- | --- |
| Echocardiography |  |  |
| RVED/LVED | 0.624 | <0.001 |
| TRV, m/s | 0.364 | <0.001 |
| Hemodynamics |  |  |
| S_v_O_2_, % | -0.480 | <0.001 |
| mRAP, mmHg | 0.232 | 0.002 |
| mPAP, mmHg | 0.409 | <0.001 |
| CI, L/min/m^2^ | -0.487 | <0.001 |
| PVR, wood units | 0.583 | <0.001 |

CI, cardiac index; LVED, left ventricular end-diastolic diameter; mPAP, mean pulmonary artery pressure; mRAP, mean right atrial pressure; PVR, pulmonary vascular resistance; RVED, right ventricular end-diastolic diameter; S_v_O_2_, mixed venous oxygen saturation; TRV, tricuspid regurgitation velocity.

Table S14 Correlation Between the Hybrid COMPERA 3-stratum Model and Echocardiographic/ Hemodynamic Parameters.

| Variables | Spearman Correlation | *P-*value |
| --- | --- | --- |
| Echocardiography |  |  |
| RVED/LVED | 0.620 | <0.001 |
| TRV, m/s | 0.408 | <0.001 |
| Hemodynamics |  |  |
| S_v_O_2_, % | -0.437 | <0.001 |
| mRAP, mmHg | 0.190 | 0.012 |
| mPAP, mmHg | 0.465 | <0.001 |
| CI, L/min/m^2^ | -0.472 | <0.001 |
| PVR, wood units | 0.637 | <0.001 |

CI, cardiac index; LVED, left ventricular end-diastolic diameter; mPAP, mean pulmonary artery pressure; mRAP, mean right atrial pressure; PVR, pulmonary vascular resistance; RVED, right ventricular end-diastolic diameter; S_v_O_2_, mixed venous oxygen saturation; TRV, tricuspid regurgitation velocity.

Table S15 The 1-, 2- and 3-year Cumulative Clinical Worsening-free Survival Rates After the First BPA Session for Each Stratum

|  | 1-year cumulative  clinical worsening-free survival rates | 2-year cumulative  clinical worsening-free survival rates | 3-year cumulative  clinical worsening-free survival rates |
| --- | --- | --- | --- |
| **The original 3-stratum model** |  |  |  |
| Low risk | 98% | 98% | 98% |
| Intermediate risk | 88% | 79% | 68% |
| High risk | 83% | 83% | 55% |
| **The hybrid 3-stratum model** |  |  |  |
| Low risk | 98% | 98% | 98% |
| Intermediate risk | 95% | 86% | 81% |
| High risk | 90% | 83% | 53% |
| **The modified 3-stratum model** |  |  |  |
| Low risk | 93% | 93% | 93% |
| Intermediate risk | 96% | 91% | 87% |
| High risk | 81% | 73% | 53% |
|  |  |  |  |
| **The original 4-stratum model** |  |  |  |
| Low risk | 97% | 97% | 97% |
| Intermediate-low risk | 100% | 88% | 88% |
| Intermediate-high risk | 82% | 75% | 60% |
| High risk | 83% | 83% | 55% |
| **The hybrid 4-stratum model** |  |  |  |
| Low risk | 97% | 97% | 97% |
| Intermediate-low risk | 100% | 88% | 88% |
| Intermediate-high risk | 90% | 86% | 80% |
| High risk | 72% | 66% | 33% |
| **The modified 4-stratum model** |  |  |  |
| Low risk | 93% | 93% | 93% |
| Intermediate-low risk | 100% | 90% | 90% |
| Intermediate-high risk | 93% | 90% | 86% |
| High risk | 72% | 66% | 33% |

BPA, balloon pulmonary angioplasty.

Table S16 Univariate Cox Analysis of Predictive Variables of Clinical Worsening.

| Variables | HR | 95% CI | *P*-value |
| --- | --- | --- | --- |
| Age | 1.053 | 1.006-1.102 | 0.028 |
| Body mass index | 1.018 | 0.910-1.139 | 0.759 |
| Sex | 0.710 | 0.328-1.535 | 0.383 |
| Disease duration | 1.077 | 1.003-1.157 | 0.042 |
| WHO-FC | 3.474 | 1.644-7.342 | 0.001 |
| Ln(NT-proBNP) | 1.940 | 1.308-2.878 | 0.001 |
| 6MWD | 0.995 | 0.992-0.998 | 0.002 |
| PH specific medicine | 1.091 | 0.581-2.050 | 0.785 |
| RVED/LVED | 6.338 | 1.908-21.058 | 0.003 |
| EF | 0.985 | 0.924-1.051 | 0.655 |
| TRV | 1.121 | 0.619-2.030 | 0.707 |
| S_v_O_2_ | 0.956 | 0.929-0.983 | 0.002 |
| mRAP | 1.117 | 1.019-1.224 | 0.018 |
| mPAP | 1.000 | 0.967-1.033 | 0.978 |
| PAWP | 1.060 | 0.868-1.295 | 0.566 |
| CI | 0.435 | 0.227-0.833 | 0.012 |
| PVR | 1.045 | 0.958-1.141 | 0.320 |
| Number of BPA sessions^a^ | 0.454 | 0.305-0.677 | <0.001 |
| Number of dilated pulmonary vessels^a^ | 0.914 | 0.873-0.958 | <0.001 |
| Original 4-stratum risk score before the 1^st^ BPA | *P* for trend < 0.001 | | |
| Low | 1 (reference) |  |  |
| Intermediate-low | 2.411 | 0.251-23.197 | 0.446 |
| Intermediate-high | 10.579 | 1.411-79.288 | 0.022 |
| High | 12.524 | 1.397-112.234 | 0.024 |
| Original 4-stratum risk score after the 1^st^ BPA | *P* for trend < 0.001 | | |
| Low | 1 (reference) |  |  |
| Intermediate-low | 3.376 | 1.057-10.777 | 0.040 |
| Intermediate-high | 14.716 | 4.709-45.989 | <0.001 |
| High | NA |  |  |
| Hybrid 4-stratum risk score before the 1^st^ BPA | *P* for trend < 0.001 | | |
| Low | 1 (reference) |  |  |
| Intermediate-low | 2.418 | 0.251-23.264 | 0.445 |
| Intermediate-high | 5.162 | 0.612-42.911 | 0.129 |
| High | 18.661 | 2.471-140.916 | 0.005 |
| Hybrid 4-stratum risk score after the 1^st^ BPA | *P* for trend < 0.001 | | |
| Low | 1 (reference) |  |  |
| Intermediate-low | 3.381 | 1.059-10.795 | 0.040 |
| Intermediate-high | 15.346 | 4.684-50.283 | <0.001 |
| High | 9.113 | 2.021-41.100 | 0.004 |

^a^, The cumulative number of BPA session/treated pulmonary vessels before the occurrence of the outcome or the end of follow-up.

BPA, balloon pulmonary angioplasty; CI: cardiac index; EF, ejection fraction; LVED, left ventricular end-diastolic diameter; mRAP: mean right atrial pressure; mPAP: mean pulmonary artery pressure; NT-proBNP, N-terminal pro-brain natriuretic peptide; PAWP, pulmonary artery wedge pressure; PVR, pulmonary vascular resistance; RVED, right ventricular end-diastolic diameter; SvO_2_, mixed venous oxygen saturation; 6MWD, 6-minute walk distance; TRV, tricuspid regurgitation velocity; WHO-FC, World Health Organization function class.

Table S17 Multivariate Cox Analysis of Predictive Variables of Clinical Worsening.

| Variables | HR | 95% CI | *P*-value |
| --- | --- | --- | --- |
| **Before the first BPA session** |  |  |  |
| Model 1 |  |  |  |
| Original COMPERA 2.0 risk score before the 1^st^ BPA | *P* for trend = 0.001 | | |
| Low | 1 (reference) |  |  |
| Intermediate-low | 1.771 | 0.181-17.312 | 0.623 |
| Intermediate-high | 8.317 | 1.098-62.975 | 0.040 |
| High | 9.726 | 1.073-88.175 | 0.043 |
| Age | 1.048 | 0.997-1.101 | 0.064 |
| Model 2 |  |  |  |
| Original COMPERA 2.0 risk score before the 1^st^ BPA | *P* for trend =0.001 | | |
| Low | 1 (reference) |  |  |
| Intermediate-low | 2.168 | 0.223-21.068 | 0.505 |
| Intermediate-high | 9.152 | 1.200-69.817 | 0.033 |
| High | 10.878 | 1.197-98.893 | 0.034 |
| Disease duration | 1.049 | 0.971-1.133 | 0.226 |
| Model 3 |  |  |  |
| Original COMPERA 2.0 risk score before the 1^st^ BPA | *P* for trend < 0.001 | | |
| Low | 1 (reference) |  |  |
| Intermediate-low | 2.425 | 0.251-23.383 | 0.444 |
| Intermediate-high | 10.652 | 1.415-80.187 | 0.022 |
| High | 12.611 | 1.402-113.456 | 0.024 |
| PH specific medicine | 0.965 | 0.490-1.900 | 0.917 |
| Model 4 |  |  |  |
| Original COMPERA 2.0 risk score before the 1^st^ BPA | *P* for trend = 0.006 | | |
| Low | 1 (reference) |  |  |
| Intermediate-low | 2.270 | 0.235-21.943 | 0.479 |
| Intermediate-high | 8.626 | 1.075-69.195 | 0.043 |
| High | 10.139 | 1.031-99.719 | 0.047 |
| RVED/LVED | 1.902 | 0.389-9.299 | 0.427 |
| Model 5 |  |  |  |
| Original COMPERA 2.0 risk score before the 1^st^ BPA | *P* for trend = 0.002 | | |
| Low | 1 (reference) |  |  |
| Intermediate-low | 2.060 | 0.208-20.411 | 0.537 |
| Intermediate-high | 8.290 | 1.088-63.183 | 0.041 |
| High | 8.739 | 0.942-81.106 | 0.057 |
| S_v_O_2_ | 0.960 | 0.920-1.001 | 0.058 |
| Model 6 |  |  |  |
| Original COMPERA 2.0 risk score before the 1^st^ BPA | *P* for trend = 0.003 | | |
| Low | 1 (reference) |  |  |
| Intermediate-low | 2.396 | 0.249-23.048 | 0.449 |
| Intermediate-high | 9.499 | 1.254-71.959 | 0.029 |
| High | 9.726 | 1.023-92.427 | 0.048 |
| mRAP | 1.055 | 0.955-1.166 | 0.292 |
| Model 7 |  |  |  |
| Original COMPERA 2.0 risk score before the 1^st^ BPA | *P* for trend = 0.005 | | |
| Low | 1 (reference) |  |  |
| Intermediate-low | 2.205 | 0.227-21.431 | 0.496 |
| Intermediate-high | 8.657 | 1.089-68.835 | 0.041 |
| High | 9.716 | 0.989-95.486 | 0.051 |
| CI | 0.752 | 0.376-1.504 | 0.420 |
| Model 8 |  |  |  |
| Original COMPERA 2.0 risk score before the 1^st^ BPA | *P* for trend = 0.002 | | |
| Low | 1 (reference) |  |  |
| Intermediate-low | 2.567 | 0.266-24.762 | 0.415 |
| Intermediate-high | 8.449 | 1.122-63.594 | 0.038 |
| High | 10.922 | 1.213-98.371 | 0.033 |
| Number of BPA sessions^a^ | 0.490 | 0.333-0.721 | <0.001 |
| Model 9 |  |  |  |
| Original COMPERA 2.0 risk score before the 1^st^ BPA | *P* for trend = 0.003 | | |
| Low | 1 (reference) |  |  |
| Intermediate-low | 2.509 | 0.261-24.168 | 0.426 |
| Intermediate-high | 7.901 | 1.044-59.771 | 0.045 |
| High | 10.385 | 1.157-93.232 | 0.037 |
| Number of dilated pulmonary vessels^a^ | 0.926 | 0.886-0.968 | 0.001 |
| Model 10 |  |  |  |
| Hybrid COMPERA 2.0 risk score before the 1^st^ BPA | *P* for trend < 0.001 | | |
| Low | 1 (reference) |  |  |
| Intermediate-low | 1.890 | 0.193-18.536 | 0.585 |
| Intermediate-high | 4.502 | 0.538-37.664 | 0.165 |
| High | 14.286 | 1.847-110.497 | 0.011 |
| Age | 1.036 | 0.987-1.088 | 0.153 |
| Model 11 |  |  |  |
| Hybrid COMPERA 2.0 risk score before the 1^st^ BPA | *P* for trend < 0.001 | | |
| Low | 1 (reference) |  |  |
| Intermediate-low | 2.185 | 0.225-21.236 | 0.500 |
| Intermediate-high | 4.505 | 0.533-38.099 | 0.167 |
| High | 16.165 | 2.102-124.340 | 0.008 |
| Disease duration | 1.048 | 0.967-1.135 | 0.253 |
| Model 12 |  |  |  |
| Hybrid COMPERA 2.0 risk score before the 1^st^ BPA | *P* for trend < 0.001 | | |
| Low | 1 (reference) |  |  |
| Intermediate-low | 2.432 | 0.252-23.455 | 0.442 |
| Intermediate-high | 5.198 | 0.623-43.390 | 0.128 |
| High | 18.789 | 2.478-142.465 | 0.005 |
| PH specific medicine | 0.965 | 0.494-1.884 | 0.917 |
| Model 13 |  |  |  |
| Hybrid COMPERA 2.0 risk score before the 1^st^ BPA | *P* for trend < 0.001 | | |
| Low | 1 (reference) |  |  |
| Intermediate-low | 2.388 | 0.247-23.114 | 0.452 |
| Intermediate-high | 4.984 | 0.567-43.845 | 0.148 |
| High | 18.029 | 2.121-153.272 | 0.008 |
| RVED/LVED | 1.138 | 0.186-6.957 | 0.889 |
| Model 14 |  |  |  |
| Hybrid COMPERA 2.0 risk score before the 1^st^ BPA | *P* for trend < 0.001 | | |
| Low | 1 (reference) |  |  |
| Intermediate-low | 2.177 | 0.222-21.311 | 0.504 |
| Intermediate-high | 4.311 | 0.511-36.372 | 0.179 |
| High | 14.431 | 1.850-112.570 | 0.011 |
| S_v_O_2_ | 0.967 | 0.924-1.012 | 0.151 |
| Model 15 |  |  |  |
| Hybrid COMPERA 2.0 risk score before the 1^st^ BPA | *P* for trend < 0.001 | | |
| Low | 1 (reference) |  |  |
| Intermediate-low | 2.402 | 0.250-23.106 | 0.448 |
| Intermediate-high | 4.608 | 0.548-38.774 | 0.160 |
| High | 16.473 | 2.145-126.493 | 0.007 |
| mRAP | 1.050 | 0.956-1.154 | 0.309 |
| Model 16 |  |  |  |
| Hybrid COMPERA 2.0 risk score before the 1^st^ BPA | *P* for trend < 0.001 | | |
| Low | 1 (reference) |  |  |
| Intermediate-low | 2.261 | 0.233-21.963 | 0.482 |
| Intermediate-high | 4.456 | 0.510-38.919 | 0.177 |
| High | 15.802 | 1.955-127.706 | 0.010 |
| CI | 0.808 | 0.415-1.571 | 0.529 |
| Model 17 |  |  |  |
| Hybrid COMPERA 2.0 risk score before the 1^st^ BPA | *P* for trend < 0.001 | | |
| Low | 1 (reference) |  |  |
| Intermediate-low | 2.571 | 0.266-24.819 | 0.414 |
| Intermediate-high | 4.217 | 0.492-36.180 | 0.189 |
| High | 13.807 | 1.817-104.906 | 0.011 |
| Number of BPA sessions^a^ | 0.528 | 0.360-0.775 | 0.001 |
| Model 18 |  |  |  |
| Hybrid COMPERA 2.0 risk score before the 1^st^ BPA | *P* for trend = 0.001 | | |
| Low | 1 (reference) |  |  |
| Intermediate-low | 2.499 | 0.259-24.069 | 0.428 |
| Intermediate-high | 4.220 | 0.492-36.160 | 0.189 |
| High | 12.852 | 1.678-98.449 | 0.014 |
| Number of dilated pulmonary vessels^a^ | 0.938 | 0.899-0.980 | 0.004 |
| **After the first BPA session** |  |  |  |
| Model 19 |  |  |  |
| Original COMPERA 2.0 risk score after the 1^st^ BPA | *P* for trend < 0.001 | | |
| Low | 1 (reference) |  |  |
| Intermediate-low | 2.971 | 0.926-9.533 | 0.067 |
| Intermediate-high | 14.355 | 4.571-45.084 | <0.001 |
| High | NA |  |  |
| Age | 1.049 | 1.003-1.097 | 0.039 |
| Model 20 |  |  |  |
| Original COMPERA 2.0 risk score after the 1^st^ BPA | *P* for trend < 0.001 | | |
| Low | 1 (reference) |  |  |
| Intermediate-low | 3.350 | 1.048-10.712 | 0.041 |
| Intermediate-high | 14.024 | 4.228-46.522 | <0.001 |
| High | NA |  |  |
| Disease duration | 1.011 | 0.932-1.096 | 0.799 |
| Model 21 |  |  |  |
| Original COMPERA 2.0 risk score after the 1^st^ BPA | *P* for trend < 0.001 | | |
| Low | 1 (reference) |  |  |
| Intermediate-low | 3.629 | 1.129-11.670 | 0.031 |
| Intermediate-high | 17.593 | 5.368-57.655 | <0.001 |
| High | NA |  |  |
| PH specific medicine | 0.678 | 0.336-1.368 | 0.278 |
| Model 22 |  |  |  |
| Original COMPERA 2.0 risk score after the 1^st^ BPA | *P* for trend < 0.001 | | |
| Low | 1 (reference) |  |  |
| Intermediate-low | 3.372 | 0.977-10.961 | 0.055 |
| Intermediate-high | 13.974 | 3.920-49.816 | <0.001 |
| High | NA |  |  |
| RVED/LVED | 1.151 | 0.256-5.169 | 0.855 |
| Model 23 |  |  |  |
| Original COMPERA 2.0 risk score after the 1^st^ BPA | *P* for trend < 0.001 | | |
| Low | 1 (reference) |  |  |
| Intermediate-low | 3.045 | 0.924-10.039 | 0.067 |
| Intermediate-high | 12.300 | 3.630-41.674 | <0.001 |
| High | NA |  |  |
| S_v_O_2_ | 0.981 | 0.936-1.027 | 0.410 |
| Model 24 |  |  |  |
| Original COMPERA 2.0 risk score after the 1^st^ BPA | *P* for trend < 0.001 | | |
| Low | 1 (reference) |  |  |
| Intermediate-low | 3.308 | 1.031-10.614 | 0.044 |
| Intermediate-high | 13.567 | 3.995-46.073 | <0.001 |
| High | NA |  |  |
| mRAP | 1.020 | 0.917-1.134 | 0.715 |
| Model 25 |  |  |  |
| Original COMPERA 2.0 risk score after the 1^st^ BPA | *P* for trend < 0.001 | | |
| Low | 1 (reference) |  |  |
| Intermediate-low | 3.196 | 0.963-10.602 | 0.058 |
| Intermediate-high | 13.333 | 3.775-47.085 | <0.001 |
| High | NA |  |  |
| CI | 0.880 | 0.435-1.778 | 0.721 |
| Model 26 |  |  |  |
| Original COMPERA 2.0 risk score after the 1^st^ BPA | *P* for trend < 0.001 | | |
| Low | 1 (reference) |  |  |
| Intermediate-low | 2.378 | 0.725-7.798 | 0.153 |
| Intermediate-high | 9.384 | 2.895-30.414 | <0.001 |
| High | NA |  |  |
| Number of BPA sessions ^a^ | 0.557 | 0.382-0.812 | 0.002 |
| Model 27 |  |  |  |
| Hybrid COMPERA 2.0 risk score after the 1^st^ BPA | *P* for trend < 0.001 | | |
| Low | 1 (reference) |  |  |
| Intermediate-low | 2.319 | 0.699-7.691 | 0.169 |
| Intermediate-high | 8.766 | 2.664-28.851 | <0.001 |
| High | NA |  |  |
| Number of dilated pulmonary vessels ^a^ | 0.940 | 0.899-0.983 | 0.006 |
| Model 28 |  |  |  |
| Hybrid COMPERA 2.0 risk score after the 1^st^ BPA | *P* for trend < 0.001 | | |
| Low | 1 (reference) |  |  |
| Intermediate-low | 3.009 | 0.938-9.654 | 0.064 |
| Intermediate-high | 14.907 | 4.507-49.298 | <0.001 |
| High | 8.331 | 1.846-37.604 | 0.006 |
| Age | 1.044 | 1.000-1.090 | 0.052 |
| Model 29 |  |  |  |
| Hybrid COMPERA 2.0 risk score after the 1^st^ BPA | *P* for trend < 0.001 | | |
| Low | 1 (reference) |  |  |
| Intermediate-low | 3.353 | 1.049-10.722 | 0.041 |
| Intermediate-high | 14.580 | 4.204-50.565 | <0.001 |
| High | 8.633 | 1.824-40.870 | 0.007 |
| Disease duration | 1.011 | 0.932-1.098 | 0.784 |
| Model 30 |  |  |  |
| Hybrid COMPERA 2.0 risk score after the 1^st^ BPA | *P* for trend < 0.001 | | |
| Low | 1 (reference) |  |  |
| Intermediate-low | 3.721 | 1.155-11.987 | 0.028 |
| Intermediate-high | 20.868 | 5.697-76.444 | <0.001 |
| High | 10.360 | 2.273-47.217 | 0.003 |
| PH specific medicine | 0.601 | 0.273-1.320 | 0.205 |
| Model 31 |  |  |  |
| Hybrid COMPERA 2.0 risk score after the 1^st^ BPA | *P* for trend < 0.001 | | |
| Low | 1 (reference) |  |  |
| Intermediate-low | 3.114 | 0.922-10.517 | 0.067 |
| Intermediate-high | 13.863 | 3.883-49.500 | <0.001 |
| High | 7.953 | 1.362-46.446 | 0.021 |
| RVED/LVED | 1.436 | 0.292-7.077 | 0.656 |
| Model 32 |  |  |  |
| Hybrid COMPERA 2.0 risk score after the 1^st^ BPA | *P* for trend < 0.001 | | |
| Low | 1 (reference) |  |  |
| Intermediate-low | 2.975 | 0.902-9.815 | 0.073 |
| Intermediate-high | 12.686 | 3.661-43.960 | <0.001 |
| High | 7.152 | 1.483-34.498 | 0.014 |
| S_v_O_2_ | 0.976 | 0.934-1.021 | 0.294 |
| Model 33 |  |  |  |
| Hybrid COMPERA 2.0 risk score after the 1^st^ BPA | *P* for trend < 0.001 | | |
| Low | 1 (reference) |  |  |
| Intermediate-low | 3.308 | 1.031-10.610 | 0.044 |
| Intermediate-high | 14.212 | 4.094-49.339 | <0.001 |
| High | 8.093 | 1.613-40.608 | 0.011 |
| mRAP | 1.022 | 0.922-1.133 | 0.683 |
| Model 34 |  |  |  |
| Hybrid COMPERA 2.0 risk score after the 1^st^ BPA | *P* for trend < 0.001 | | |
| Low | 1 (reference) |  |  |
| Intermediate-low | 3.131 | 0.944-10.388 | 0.062 |
| Intermediate-high | 13.607 | 3.791-48.841 | <0.001 |
| High | 7.700 | 1.491-39.756 | 0.015 |
| CI | 0.835 | 0.414-1.687 | 0.616 |
| Model 35 |  |  |  |
| Hybrid COMPERA 2.0 risk score after the 1^st^ BPA | *P* for trend < 0.001 | | |
| Low | 1 (reference) |  |  |
| Intermediate-low | 2.364 | 0.721-7.750 | 0.156 |
| Intermediate-high | 10.658 | 3.153-36.026 | <0.001 |
| High | 5.787 | 1.254-26.699 | 0.024 |
| Number of BPA sessions ^a^ | 0.541 | 0.372-0.786 | 0.001 |
| Model 36 |  |  |  |
| Hybrid COMPERA 2.0 risk score after the 1^st^ BPA | *P* for trend < 0.001 | | |
| Low | 1 (reference) |  |  |
| Intermediate-low | 2.295 | 0.692-7.611 | 0.175 |
| Intermediate-high | 8.971 | 2.587-31.105 | 0.001 |
| High | 6.342 | 1.387-28.999 | 0.017 |
| Number of dilated pulmonary vessels ^a^ | 0.937 | 0.897-0.980 | 0.004 |

^a^, The cumulative number of BPA session/treated pulmonary vessels before the occurrence of the outcome or the end of follow-up.

BPA, balloon pulmonary angioplasty; CI: cardiac index; LVED, left ventricular end-diastolic diameter; mRAP: mean right atrial pressure; RVED, right ventricular end-diastolic diameter; SvO_2_, mixed venous oxygen saturation.

Table S18 Change in Risk Stratum after the 1^st^ BPA Session by Risk at Baseline (the Original 3-stratum Model)

| Risk | Low after the 1^st^ BPA | Intermediate after the 1^st^ BPA | High after the 1^st^ BPA | Sum |
| --- | --- | --- | --- | --- |
| Low before the 1^st^ BPA | 48 | 0 | 0 | 48 |
| Intermediate before the 1^st^ BPA | 45 | 70 | 0 | 115 |
| High before the 1^st^ BPA | 0 | 9 | 3 | 12 |
| Sum | 93 | 79 | 3 | 175 |

BPA, balloon pulmonary angioplasty.

Table S19 Change in Risk Stratum after the 1^st^ BPA Session by Risk at Baseline (Original 4-stratum Model)

| Risk | Low after the 1^st^ BPA | Intermediate-low after the 1^st^ BPA | Intermediate-high after the 1^st^ BPA | High after the 1^st^ BPA | Sum |
| --- | --- | --- | --- | --- | --- |
| Low before the 1^st^ BPA | 35 | 2 | 0 | 0 | 37 |
| Intermediate-low before the 1^st^ BPA | 34 | 18 | 0 | 0 | 52 |
| Intermediate-high before the 1^st^ BPA | 8 | 47 | 19 | 0 | 74 |
| High before the 1^st^ BPA | 0 | 3 | 6 | 3 | 12 |
| Sum | 77 | 70 | 25 | 3 | 175 |

BPA, balloon pulmonary angioplasty.

Table S20 Change in Risk Stratum after the 1^st^ BPA Session by Risk at Baseline (the Hybrid 3-stratum Model)

| Risk | Low after the 1^st^ BPA | Intermediate after the 1^st^ BPA | High after the 1^st^ BPA | Sum |
| --- | --- | --- | --- | --- |
| Low before the 1^st^ BPA | 48 | 0 | 0 | 48 |
| Intermediate before the 1^st^ BPA | 37 | 28 | 0 | 65 |
| High before the 1^st^ BPA | 8 | 40 | 14 | 62 |
| Sum | 93 | 68 | 14 | 175 |

BPA, balloon pulmonary angioplasty.

Table S21 Change in Risk Stratum after the 1^st^ BPA Session by Risk at Baseline with (Hybrid 4-stratum Model)

| Risk | Low after the 1^st^ BPA | Intermediate-low after the 1^st^ BPA | Intermediate-high after the 1^st^ BPA | High after the 1^st^ BPA | Sum |
| --- | --- | --- | --- | --- | --- |
| Low before the 1^st^ BPA | 35 | 2 | 0 | 0 | 37 |
| Intermediate-low before the 1^st^ BPA | 34 | 18 | 0 | 0 | 52 |
| Intermediate-high before the 1^st^ BPA | 8 | 32 | 8 | 0 | 48 |
| High before the 1^st^ BPA | 0 | 18 | 12 | 8 | 38 |
| Sum | 77 | 70 | 20 | 8 | 175 |

BPA, balloon pulmonary angioplasty.

Table S22 Univariate Logistic Analysis of Variables in Predicating Reaching Low Risk after the First BPA Session or during Subsequent Sessions^a^.

| Variable | OR | 95% CI | *P*-value |
| --- | --- | --- | --- |
| Age | 0.969 | 0.933-1.007 | 0.105 |
| Body mass index | 1.071 | 0.961-1.192 | 0.214 |
| Sex | 0.968 | 0.487-1.926 | 0.927 |
| Disease duration | 0.943 | 0.877-1.014 | 0.111 |
| WHO-FC | 0.450 | 0.194-1.045 | 0.063 |
| Ln(NT-proBNP) | 0.373 | 0.239-0.583 | <0.001 |
| 6MWD | 1.007 | 1.004-1.011 | <0.001 |
| PH specific medicine | 0.570 | 0.297-1.092 | 0.090 |
| RVED/LVED | 0.020 | 0.003-0.127 | <0.001 |
| EF | 0.987 | 0.935-1.043 | 0.648 |
| TRV | 0.919 | 0.506-1.668 | 0.780 |
| S_v_O_2_ | 1.082 | 1.027-1.141 | 0.003 |
| mRAP | 0.911 | 0.828-1.002 | 0.055 |
| mPAP | 0.996 | 0.967-1.026 | 0.789 |
| PAWP | 0.873 | 0.749-1.018 | 0.084 |
| CI | 2.742 | 1.427-5.269 | 0.002 |
| PVR | 0.921 | 0.849-0.998 | 0.045 |
| Number of BPA sessions^b^ | 0.797 | 0.584-1.087 | 0.151 |
| Number of dilated segmental pulmonary arteries^b^ | 1.008 | 0.969-1.049 | 0.681 |
| Time interval | 0.977 | 0.921-1.036 | 0.434 |
| Original COMPERA 2.0 risk score before the 1^st^ BPA | *P* for trend < 0.001 | | |
| Intermediate-low | 1 (reference) |  |  |
| Intermediate-high | 0.209 | 0.089-0.490 | <0.001 |
| High | 0.105 | 0.026-0.424 | 0.002 |
| Hybrid COMPERA 2.0 risk score before the 1^st^ BPA | *P* for trend < 0.001 | | |
| Intermediate-low | 1 (reference) |  |  |
| Intermediate-high | 0.382 | 0.150-0.968 | 0.043 |
| High | 0.075 | 0.027-0.207 | <0.001 |

^a^, Only patients with intermediate-low risk or higher grade at baseline were included in the analysis.

^b^, The cumulative number of BPA session/treated pulmonary vessels before the occurrence of the outcome.

BPA, balloon pulmonary angioplasty; CI: cardiac index; EF, ejection fraction; LVED, left ventricular end-diastolic diameter; mRAP: mean right atrial pressure; mPAP: mean pulmonary artery pressure; NT-proBNP, N-terminal pro-brain natriuretic peptide; PAWP, pulmonary artery wedge pressure; PVR, pulmonary vascular resistance; RVED, right ventricular end-diastolic diameter; SvO_2_, mixed venous oxygen saturation; 6MWD, 6-minute walk distance; TRV, tricuspid regurgitation velocity; WHO-FC, World Health Organization function class.

Table S23 Multivariate Logistic Analysis of Variables in Predicating Reaching Low Risk after the First BPA Session or during Subsequent Sessions^a^.

| Variables | OR | 95% CI | *P*-value |
| --- | --- | --- | --- |
| Model 1 |  |  |  |
| Original COMPERA 2.0 risk score before the 1^st^ BPA | *P* for trend < 0.001 | | |
| Intermediate-low | 1 (reference) |  |  |
| Intermediate-high | 0.202 | 0.081-0.502 | 0.001 |
| High | 0.093 | 0.021-0.415 | 0.002 |
| Age | 0.955 | 0.915-0.997 | 0.035 |
| Disease duration | 0.948 | 0.875-1.026 | 0.188 |
| PH specific medicine | 0.586 | 0.282-1.218 | 0.152 |
| Number of BPA sessions^b^ | 0.971 | 0.678-1.389 | 0.871 |
| Model 2 |  |  |  |
| Original COMPERA 2.0 risk score before the 1^st^ BPA | *P* for trend = 0.068 | | |
| Intermediate-low | 1 (reference) |  |  |
| Intermediate-high | 0.407 | 0.155-1.069 | 0.068 |
| High | 0.301 | 0.058-1.554 | 0.152 |
| RVED/LVED | 0.081 | 0.010-0.624 | 0.016 |
| S_v_O_2_ | 1.031 | 0.981-1.083 | 0.228 |
| mRAP | 1.004 | 0.896-1.126 | 0.945 |
| CI | 1.250 | 0.555-2.819 | 0.590 |
| Model 3 |  |  |  |
| Original COMPERA 2.0 risk score before the 1^st^ BPA | *P* for trend = 0.045 | | |
| Intermediate-low | 1 (reference) |  |  |
| Intermediate-high | 0.341 | 0.125-0.933 | 0.036 |
| High | 0.270 | 0.053-1.385 | 0.117 |
| RVED/LVED | 0.070 | 0.008-0.583 | 0.014 |
| S_v_O_2_ | 1.034 | 0.986-1.085 | 0.165 |
| PAWP | 0.938 | 0.784-1.121 | 0.480 |
| PVR | 1.038 | 0.931-1.156 | 0.506 |
| Model 4 |  |  |  |
| Hybrid COMPERA 2.0 risk score before the 1^st^ BPA | *P* for trend < 0.001 | | |
| Intermediate-low | 1 (reference) |  |  |
| Intermediate-high | 0.337 | 0.126-0.900 | 0.030 |
| High | 0.070 | 0.023-0.217 | 0.070 |
| Age | 0.970 | 0.928-1.014 | 0.172 |
| Disease duration | 0.952 | 0.875-1.035 | 0.250 |
| PH specific medicine | 0.561 | 0.263-1.195 | 0.134 |
| Number of BPA sessions^b^ | 1.107 | 0.747-1.641 | 0.613 |
| Model 5 |  |  |  |
| Hybrid COMPERA 2.0 risk score before the 1^st^ BPA | *P* for trend = 0.002 | | |
| Intermediate-low | 1 (reference) |  |  |
| Intermediate-high | 0.679 | 0.237-1.944 | 0.470 |
| High | 0.165 | 0.052-0.522 | 0.002 |
| RVED/LVED | 0.131 | 0.017-1.024 | 0.053 |
| S_v_O_2_ | 1.029 | 0.979-1.082 | 0.264 |
| mRAP | 0.989 | 0.880-1.112 | 0.0854 |
| CI | 1.298 | 0.563-2.995 | 0.541 |
| Model 6 |  |  |  |
| Hybrid COMPERA 2.0 risk score before the 1^st^ BPA | *P* for trend = 0.001 | | |
| Intermediate-low | 1 (reference) |  |  |
| Intermediate-high | 0.560 | 0.188-1.663 | 0.296 |
| High | 0.143 | 0.044-0.469 | 0.001 |
| RVED/LVED | 0.110 | 0.013-0.921 | 0.042 |
| S_v_O_2_ | 1.033 | 0.984-1.084 | 0.195 |
| PAWP | 0.942 | 0.779-1.139 | 0.540 |
| PVR | 1.031 | 0.920-1.155 | 0.596 |

^a^, Only patients with intermediate-low risk or higher grade at baseline were included in the analysis.

^b^, The cumulative number of BPA session before the occurrence of the outcome or the end of follow-up.

BPA, balloon pulmonary angioplasty; CI: cardiac index; LVED, left ventricular end-diastolic diameter; mRAP: mean right atrial pressure; PAWP, pulmonary artery wedge pressure; PVR, pulmonary vascular resistance; RVED, right ventricular end-diastolic diameter; SvO_2_, mixed venous oxygen saturation.

Table S24 Univariate Logistic Analysis of Variables in Predicating Hemodynamic Response during Subsequent BPA Sessions^a^.

| Variables | OR | 95% CI | *P*-value |
| --- | --- | --- | --- |
| Age | 0.994 | 0.957-1.032 | 0.751 |
| Body mass index | 1.090 | 0.969-1.225 | 0.150 |
| Sex | 1.019 | 0.449-2.312 | 0.964 |
| Disease duration | 0.909 | 0.810-1.021 | 0.107 |
| WHO-FC | 0.307 | 0.135-0.701 | 0.005 |
| Ln(NT-proBNP) | 0.510 | 0.357-0.727 | <0.001 |
| 6MWD | 1.014 | 1.007-1.022 | <0.001 |
| PH specific medicine | 0.471 | 0.228-0.974 | 0.042 |
| RVED/LVED | 0.030 | 0.003-0.326 | 0.004 |
| EF | 1.025 | 0.960-1.094 | 0.464 |
| TRV | 0.282 | 0.129-0.619 | 0.002 |
| S_v_O_2_ | 1.047 | 0.979-1.119 | 0.182 |
| mRAP | 0.956 | 0.856-1.068 | 0.429 |
| mPAP | 0.926 | 0.885-0.969 | 0.001 |
| PAWP | 1.030 | 0.827-1.283 | 0.793 |
| CI | 2.229 | 1.126-4.409 | 0.021 |
| PVR | 0.807 | 0.714-0.911 | 0.001 |
| Number of BPA sessions^b^ | 1.114 | 0.829-1.498 | 0.474 |
| Number of dilated segmental pulmonary arteries^b^ |  |  |  |
| Time interval | 0.997 | 0.953-1.043 | 0.900 |
| Original COMPERA 4-stratum risk score before the 1^st^ BPA | *P* for trend < 0.001 | | |
| Low | 1 (reference) |  |  |
| Intermediate-low | 0.399 | 0.132-1.203 | 0.103 |
| Intermediate-high | 0.176 | 0.059-0.520 | 0.002 |
| High | NA^c^ |  |  |
| Hybrid COMPERA 4-stratum risk score before the 1^st^ BPA | *P* for trend <0.001 | | |
| Low | 1 (reference) |  |  |
| Intermediate-low | 0.399 | 0.132-1.203 | 0.103 |
| Intermediate-high | 0.216 | 0.070-0.663 | 0.007 |
| High | 0.046 | 0.005-0.401 | 0.005 |

^a^, Only patients with mPAP≥ 30 mmHg at baseline were included in the analysis.

^b^, The cumulative number of BPA session/treated pulmonary vessels before the occurrence of the outcome or the end of follow-up.

c, No one reached mPAP < 30 mmHg and OR could not be calculated.

BPA, balloon pulmonary angioplasty; CI: cardiac index; EF, ejection fraction; LVED, left ventricular end-diastolic diameter; mRAP: mean right atrial pressure; mPAP: mean pulmonary artery pressure; NT-proBNP, N-terminal pro-brain natriuretic peptide; PAWP, pulmonary artery wedge pressure; PVR, pulmonary vascular resistance; RVED, right ventricular end-diastolic diameter; S_v_O_2_, mixed venous oxygen saturation; 6MWD, 6-minute walk distance; TRV, tricuspid regurgitation velocity; WHO-FC, World Health Organization function class.

Table S25 Multivariate Logistic Analysis of Variables in Predicating Hemodynamic Response at Follow-up^a^.

| Variables | OR | 95% CI | *P*-value |
| --- | --- | --- | --- |
| Model 1 |  |  |  |
| Original COMPERA 2.0 risk score before the 1^st^ BPA | *P* for trend = 0.063 | | |
| Low | 1 (reference) |  |  |
| Intermediate-low | 0.587 | 0.180-1.917 | 0.378 |
| Intermediate-high | 0.388 | 0.104-1.451 | 0.159 |
| High | NA^c^ |  |  |
| Disease duration | 0.932 | 0.834-1.042 | 0.218 |
| PH specific medicine | 0.580 | 0.277-1.217 | 0.150 |
| RVED/LVED | 0.111 | 0.007-1.858 | 0.126 |
| Model 2 |  |  |  |
| Original COMPERA 2.0 risk score before the 1^st^ BPA | *P* for trend = 0.048 | | |
| Low | 1 (reference) |  |  |
| Intermediate-low | 0.477 | 0.144-1.582 | 0.226 |
| Intermediate-high | 0.354 | 0.094-1.334 | 0.125 |
| High | NA^c^ |  |  |
| TRV | 0.390 | 0.169-0.901 | 0.028 |
| S_v_O_2_ | 1.000 | 0.945-1.058 | 0.999 |
| CI | 1.474 | 0.598-3.635 | 0.400 |
| Model 3 |  |  |  |
| Original COMPERA 2.0 risk score before the 1^st^ BPA | *P* for trend = 0.019 | | |
| Low | 1 (reference) |  |  |
| Intermediate-low | 0.489 | 0.151-1.591 | 0.235 |
| Intermediate-high | 0.308 | 0.089-1.064 | 0.063 |
| High | NA^c^ |  |  |
| mPAP | 0.925 | 0.875-0.978 | 0.006 |
| mRAP | 1.057 | 0.924-1.209 | 0.421 |
| Number of BPA sessions^b^ | 1.392 | 0.970-1.998 | 0.073 |
| Model 4 |  |  |  |
| Original COMPERA 2.0 risk score before the 1^st^ BPA | *P* for trend = 0.083 | | |
| Low | 1 (reference) |  |  |
| Intermediate-low | 0.576 | 0.178-1.866 | 0.357 |
| Intermediate-high | 0.490 | 0.123-1.961 | 0.314 |
| High | NA^c^ |  |  |
| PVR | 0.817 | 0.697-0.957 | 0.012 |
| mRAP | 1.01 | 0.890-1.168 | 0.781 |
| Number of BPA sessions^b^ | 1.267 | 0.907-1.769 | 0.165 |
| Model 5 |  |  |  |
| Hybrid COMPERA 2.0 risk score before the 1^st^ BPA | *P* for trend = 0.051 | | |
| Low | 1 (reference) |  |  |
| Intermediate-low | 0.566 | 0.174-1.848 | 0.346 |
| Intermediate-high | 0.407 | 0.108-1.526 | 0.182 |
| High | 0.107 | 0.010-1.144 | 0.064 |
| Disease duration | 0.934 | 0.835-1.045 | 0.232 |
| PH specific medicine | 0.579 | 0.277-1.211 | 0.146 |
| RVED/LVED | 0.164 | 0.010-2.758 | 0.209 |
| Model 6 |  |  |  |
| Hybrid COMPERA 2.0 risk score before the 1^st^ BPA | *P* for trend = 0.035 | | |
| Low | 1 (reference) |  |  |
| Intermediate-low | 0.476 | 0.144-1.577 | 0.224 |
| Intermediate-high | 0.436 | 0.111-1.716 | 0.235 |
| High | 0.094 | 0.010-0.909 | 0.041 |
| TRV | 0.398 | 0.174-0.913 | 0.030 |
| S_v_O_2_ | 0.999 | 0.945-1.057 | 0.976 |
| CI | 1.493 | 0.599-3.719 | 0.389 |
| Model 7 |  |  |  |
| Hybrid COMPERA 2.0 risk score before the 1^st^ BPA | *P* for trend = 0.015 | | |
| Low | 1 (reference) |  |  |
| Intermediate-low | 0.484 | 0.149-1.567 | 0.226 |
| Intermediate-high | 0.381 | 0.107-1.359 | 0.137 |
| High | 0.067 | 0.007-0.684 | 0.023 |
| mPAP | 0.929 | 0.880-0.981 | 0.008 |
| mRAP | 1.035 | 0.910-1.176 | 0.604 |
| Number of BPA sessions^b^ | 1.411 | 0.980-2.031 | 0.064 |
| Model 8 |  |  |  |
| Hybrid COMPERA 2.0 risk score before the 1^st^ BPA | *P* for trend = 0.063 | | |
| Low | 1 (reference) |  |  |
| Intermediate-low | 0.567 | 0.175-1.841 | 0.345 |
| Intermediate-high | 0.632 | 0.147-2.729 | 0.539 |
| High | 0.091 | 0.009-0.972 | 0.047 |
| PVR | 0.823 | 0.701-0.967 | 0.018 |
| mRAP | 0.989 | 0.868-1.128 | 0.871 |
| Number of BPA sessions^b^ | 1.290 | 0.922-1.806 | 0.137 |

^a^, Only patients with mPAP≥ 30 mmHg at baseline were included in the analysis.

^b^, The cumulative number of BPA session before the occurrence of the outcome or the end of follow-up.

^c^, No one reached mPAP < 30 mmHg and OR could not be calculated.

BPA, balloon pulmonary angioplasty; LVED, left ventricular end-diastolic diameter; mRAP: mean right atrial pressure; mPAP: mean pulmonary artery pressure; NT-proBNP, N-terminal pro-brain natriuretic peptide; PVR, pulmonary vascular resistance; RVED, right ventricular end-diastolic diameter; S_v_O_2_, mixed venous oxygen saturation; 6MWD, 6-minute walk distance; TRV, tricuspid regurgitation velocity; WHO-FC, World Health Organization function class.
